# Identification of an autophagy-related gene signature for predicting prognosis and immune activity in pancreatic adenocarcinoma

**DOI:** 10.1038/s41598-022-11050-w

**Published:** 2022-04-29

**Authors:** Jiang Deng, Qian Zhang, Liping Lv, Ping Ma, Yangyang Zhang, Ning Zhao, Yanyu Zhang

**Affiliations:** 1 Institute of Health Service and Transfusion Medicine, Beijing, 100850 People’s Republic of China; 2 Beijing Key Laboratory of Blood Safety and Supply Technologies, Beijing, 100850 People’s Republic of China

**Keywords:** Cancer genetics, Gastrointestinal cancer, Tumour biomarkers

## Abstract

Adenocarcinoma of the pancreas (PAAD) is a cancerous growth that deteriorates rapidly and has a poor prognosis. Researchers are investigating autophagy in PAAD to identify a new biomarker and treatment target. An autophagy-related gene (ARG) model for overall survival (OS) was constructed using multivariate Cox regression analyses. A cohort of the Cancer Genome Atlas (TCGA)-PAAD was used as the training group as a basis for model construction. This prediction model was validated with several external datasets. To evaluate model performance, the analysis with receiver operating characteristic curves (ROC) was performed. The Human Protein Atlas (HPA) and Cancer Cell Line Encyclopedia (CCLE) were investigated to validate the effects of ARGs expression on cancer cells. Comparing the levels of immune infiltration between high-risk and low-risk groups was finished through the use of CIBERSORT. The differentially expressed genes (DEGs) between the low-/high-risk groups were analyzed further via Gene Ontology biological process (GO-BP) and Kyoto Encyclopedia of Genes and Genomes (KEGG) analyses, which were used to identify potential small-molecule compounds in Connectivity Map (CMap), followed by half-maximal inhibitory concentration (IC50) examination with PANC-1 cells. The risk score was finally calculated as follows: BAK1 × 0.34 + ITGA3 × 0.38 + BAG3 × 0.35 + APOL1 × 0.26–RAB24 × 0.67519. ITGA3 and RAB24 both emerged as independent prognostic factors in multivariate Cox regression. Each PAAD cohort had a significantly shorter OS in the high-risk group than in the low-risk group. The high-risk group exhibited infiltration of several immune cell types, including naive B cells (*p* = 0.003), plasma cells (*p* = 0.044), and CD8 T cells (nearly significant, *p* = 0.080). Higher infiltration levels of NK cells (*p* = 0.025), resting macrophages (*p* = 0.020), and mast cells (*p* = 0.007) were found in the high-risk group than the low-risk group. The in vitro and in vivo expression of signature ARGs was consistent in the CCLE and HPA databases. The top 3 enriched Gene Ontology biological processes (GO-BPs) were signal release, regulation of transsynaptic signaling, and modulation of chemical synaptic transmission, and the top 3 enriched Kyoto Encyclopedia of Genes and Genomes (KEGG) pathways were MAPK, cAMP, and cell adhesion molecules. Four potential small-molecule compounds (piperacetazine, vinburnine, withaferin A and hecogenin) that target ARGs were also identified. Taking the results together, our research shows that the ARG signature may serve as a useful prognostic indicator and reveal potential therapeutic targets in patients with PAAD.

## Introduction

Adenocarcinoma of the pancreas (PAAD) is a cancerous growth that deteriorates rapidly and has a poor prognosis. Due to its high mortality rate, PAAD has a low incidence rate and an almost identical mortality rate^1^. According to some studies, pancreatic cancer may overtake lung cancer by 2020 as the fourth most common cause of death from cancer in developed countries^2,3^. A significant proportion of deaths due to pancreatic cancer is caused by the lack of evident clinical symptoms in the early stages of the disease, which delays treatment^4^. It is estimated that only 20% of patients diagnosed with pancreatic cancer can undergo surgery, and the 5-year survival rate increases by only 20–30% after surgery^5,6^. Considering that the prognosis of pancreatic cancer patients is poor, it is imperative that a prognosis prediction model be developed and then treatment plans based upon it developed.

The autophagic process is a highly conserved system of degrading nonessential components within cells. The autophagy process is activated during times of metabolic stress for the purpose of providing alternative energy sources, including autophagosome formation, nucleation, double-membrane growth and closure, and fellowed by lysosomal fusion; this process helps maintain homeostasis and viability^7^. Various diseases have been linked to abnormal autophagy, including malignant tumors^8,9^. Cancer treatments such as chemotherapy^10^, targeted therapy^11^ or immunotherapy^12^ have most commonly been described as resistant to autophagy as a mechanism of resistance. Autophagy, however, appears to work in both directions to regulate tumorigenesis. Low autophagy levels promote cancer initiation in early-stage cancers, while high autophagy levels promote survival of tumor cells in nutrient-deficient environments^13,14^. It has been demonstrated that several human pancreatic ductal carcinoma cell lines exhibit high levels of autophagy, whereas no autophagy is evident in the pancreatic ducts of normal individuals^15–17^. Despite this, several reports have found that impaired autophagy promotes cancer since chronic stress, which contributes to PDAC in patients, impairs the levels of autophagy in the pancreas^18^. Therefore, it is unclear what role autophagy plays in pancreatic cancer^19,20^.

On the basis of gene expression signatures based on autophagy-related genes (ARGs), several cancers such as colon cancer^21^, breast cancer^22^, ovarian cancer^23^, and non-small-cell lung cancer^24^ have been associated with gene expression signatures. An ARG-based prognostic model for pancreatic cancer was reported in a recent study; however, the results were not confirmed with external databases^25^. To identify differentially expressed genes (DEGs), we integrated data from The Cancer Genome Atlas (TCGA) and Genotype-Tissue Expression (GTEx) databases. In the development of our prognostic model, ARGs were examined, and their performance was evaluated using multiple external datasets (GSE57495, GSE78229), GDS4336, GSE85916, and ICGC-PACA-AU), all of which are associated with pancreatic cancer. According to our research, the expression of ARGs is associated with immune infiltration of tumors, and we developed a series of small-molecule compounds that target ARGs. These results suggest that the ARG signature may offer patients with PAAD prognostic information and provide potential drug targets.

## Results

### Characteristics of the patients included in the datasets

**Figure 1:**
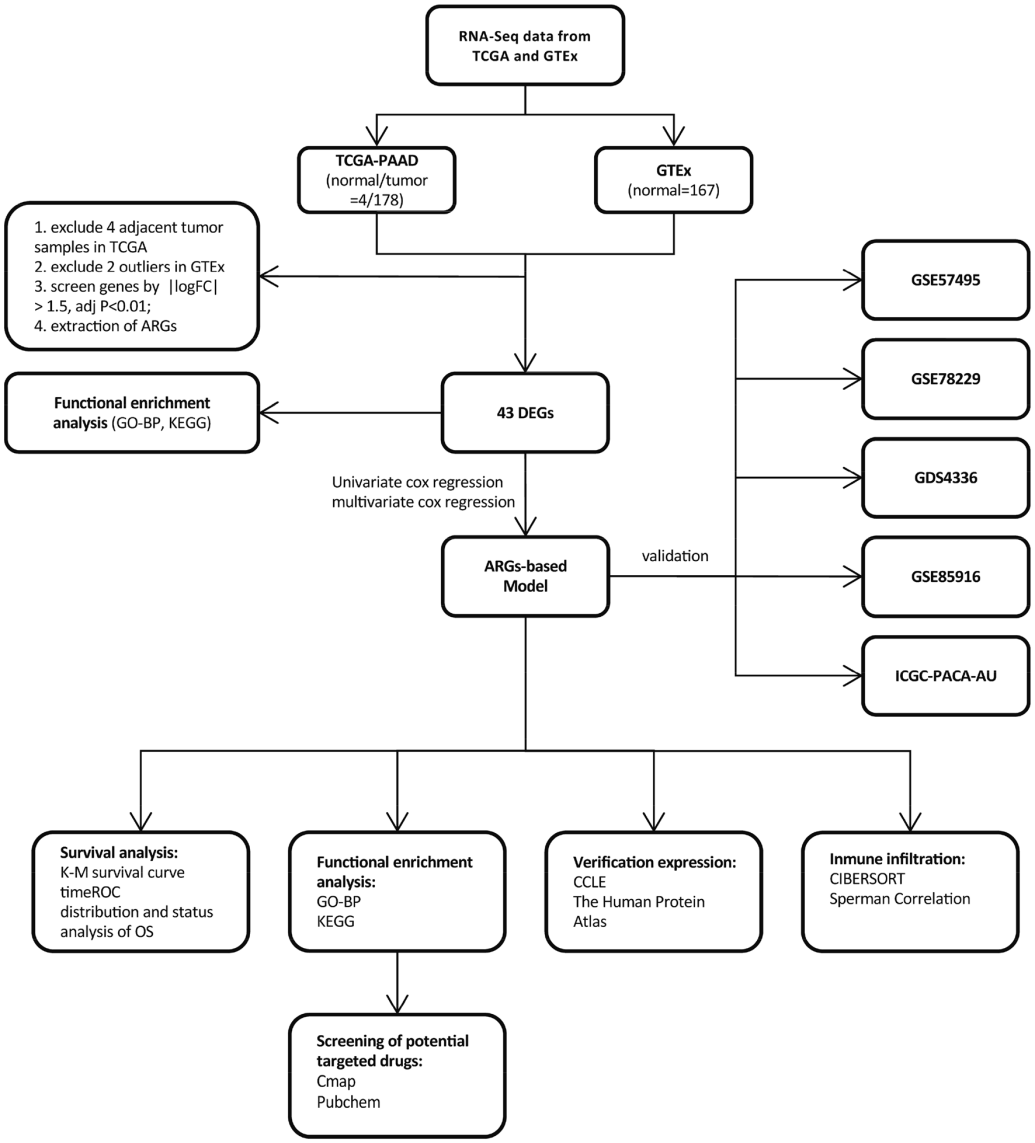
Diagram of the Overall Study Design.

The sequencing information of pancreatic cancer patients from the TCGA-PAAD database, which contains 176 tumor samples and 4 adjacent samples, was downloaded. Subsequently, we used only the 176 tumor samples because there were a small number of adjacent tumor samples. As a control group, we downloaded sequence data from the GTEx database. Following filtering, 2 tissue samples with extremely low sequencing data were excluded, and finally, 165 normal pancreatic tissue samples were used to identify DEGs. As external data sources, we used the GEO and ICGC websites to examine the clinical applicability of the signature in various databases. Table 1 shows the characteristics of each dataset (Fig. 1).

**Table 1:** Clinical data of PAAD cohort from TCGA, GDS4336, GSE57495, GSE78229, GSE85916, ICGC-PACA-AU.

	TCGA-PAAD	GDS4336	GSE57495	GSE78229	GSE85916	ICGC-PACA-AU
**Age (year)**						
< 65	81	–	–	–	–	33
≥ 65	95	–	–	–	–	46
Unknown	0	–	–	–	–	1
**Gender**						
Female	80	–	–	–	–	40
Male	96	–	–	–	–	40
**Survival status**						
Alive	88	13	21	14	22	32
Dead	88	29	42	35	57	48
**Grade**						
Grade 1	30	–	–	2	–	–
Grade 2	94	–	–	24	–	–
Grade 3	48	–	–	22	–	–
Grade 4	2	–	–	1	–	–
unknown	2	–	–	0	–	–
**Stage**						
Stage I	21	–	13	4	–	–
Stage II	145	–	50	45	–	–
Stage III	3	–	0	0	–	–
Stage IV	4	–	0	0	–	–
Unknown	3	–	0	0	–	–
**T**						
T1	7	–	–	–	–	–
T2	24	–	–	–	–	–
T3	140	–	–	–	–	–
T4	3	–	–	–	–	–
Unknown	2	–	–	–	–	–
**M**						
M0	79	–	–	–	–	–
M1	4	–	–	–	–	–
Unknown	93	–	–	–	–	–
**N**						
N0	49	–	–	–	–	–
N1	122	–	–	–	–	–
Unknown	5	–	–	–	–	–

### Identification of differentially expressed ARGs

**Figure 2:**
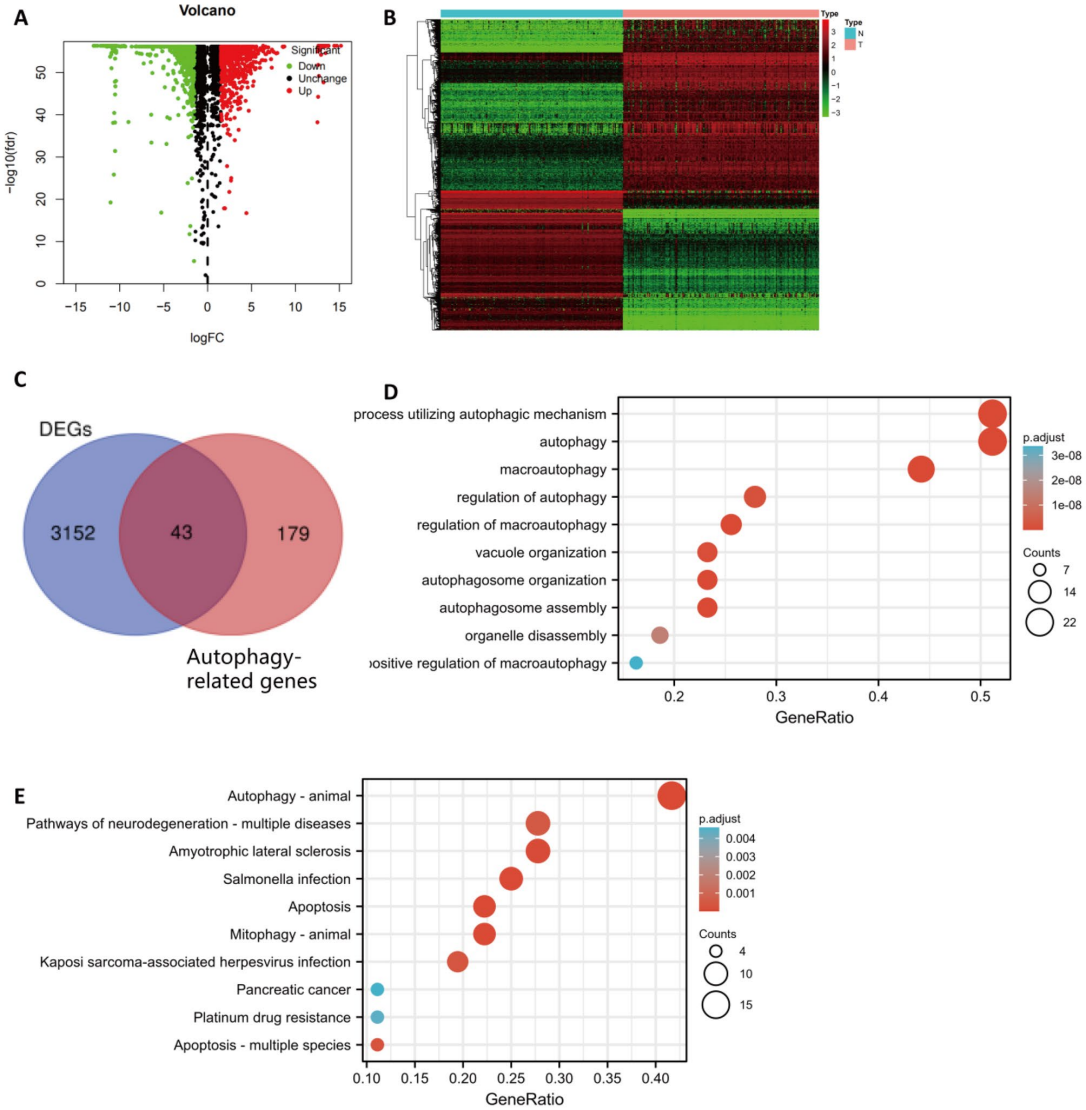
Identification and Enrichment Analysis of the Autophagy-Related DEGs. (**A**) Using the GTEx cohort data with the PAAD cohort data from the TCGA, the following volcano plot is derived. Red dots represent genes which are upregulated, green dots represent genes which are downregulated, and black dots indicate genes which are neither up- nor downregulated. (**B**) A heatmap illustrating the expression levels of DEGs. (**C**) Overlapping genes between the DEGs and ARGs. A bubble chart showing the enriched GO-BPs and KEGG pathways (**D**–**E**).

Using the normalizeBetweenArrays function in R software, we combined the TCGA-PAAD dataset with the GTEx normal pancreatic tissue dataset. Based on the criteria, a total of 3195 genes were found to be differentially expressed (Supplementary Table 1). Figure 2A–B shows the volcano and heatmaps. By overlapping the differentially expressed ARGs with the 232 ARGs from the HADb website, a total of 43 differentially expressed ARGs were selected (Fig. 2C and Supplementary Table 2).

Numerous enriched GO-BPs and KEGG pathways were examined in the functional enrichment analysis (Fig. 2D–E). Based on GO-BP analysis, the ARGs mainly functioned to regulate autophagy and to utilize autophagic mechanisms. Based on KEGG analysis, these genes were indicated to be mainly involved in signaling pathways related to autophagy-animal. Moreover, we added unbiased GO-BP and KEGG analyses to Supplementary Fig. 1 by using all the DEGs.

### Establishment of a prognostic signature with 5 ARGs

**Figure 3:**
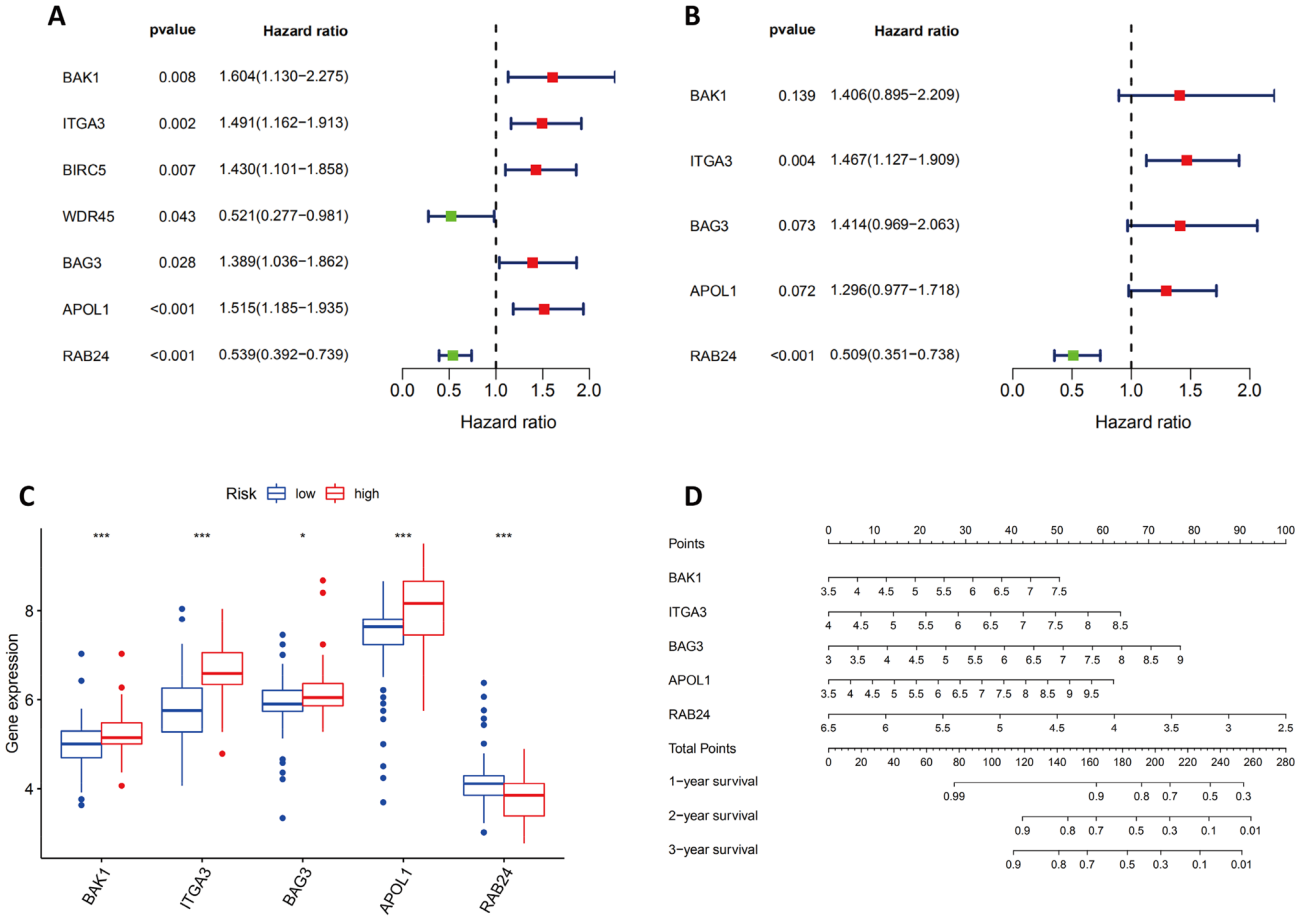
Establishment of a Prognostic Signature with 5 ARGs. Cox regression analyses of the ARG hazard ratios (HRs) in PAAD according to univariate (**A**) and multivariate (**B**) analyses. Red boxes indicate higher-risk ARGs with HRs > 1; green boxes indicate lower-risk ARGs with HRs < 1. (**C**) The levels of the 5 ARGs in the low/ high-risk groups. (**D**) A nomogram was generated to predict the survival rates of pancreatic cancer patients after one, two, and three years of therapy. A symbol of *, **, or *** denotes a *p* < 0.05, *p* < 0.01, or *p* < 0.001, respectively.

Using univariate Cox regression, a forest map was generated showing seven ARGs associated with pancreatic cancer prognosis: BAK1, ITGA3, BIRC5, WDR45, BAG3, APOL1, and RAB24 (Fig. 3A). Two of these 7 ARGs were protective, whereas 5 were associated with an increased risk. Then, we constructed a multivariate regression equation of these 7 genes, and we included 5 of them in the risk signature model. The final risk score was defined as [Expression level of BAK1 × (0.34085)] + [Expression level of ITGA3 × (0.38309)] + [Expression level of BAG3 × (0.34635)] + [Expression level of APOL1 × (0.25892)] + [Expression level of RAB24 × (−0.67519)]. Based on a multivariable Cox regression analysis, ITGA3 was identified as an independent high-risk ARG (hazard ratio [HR] = 1.47, 95% confidence interval [CI] = 1.13–1.91); RAB24 was an independent low-risk ARG (HR = 0.51, 95% CI = 0.35–0.74) (Fig. 3B).

Based upon the median risk scores of the TCGA-PAAD cohort, the patients in the TCGA dataset, and a series of external datasets (GSE57495, GSE78229, GDS4336, GSE85916, and ICGC-PACA-AU) were divided into high- and low-risk groups.

In the TCGA cohort, we examined the expression of the 5 ARGs in the two risk groups. One of the five identified ARGs (RAB24) is downregulated in the high-risk group, whereas four of them are upregulated in the high-risk group (BAK1, ITGA3, BAG3, and APOL1) (Fig. 3C). To assess the ARG risk score's ability to accurately predict patients' 1-, 2-, and 3-year survival rates we generated nomograms which were used to make this evaluation (Fig. 3D).

**Figure 4:**
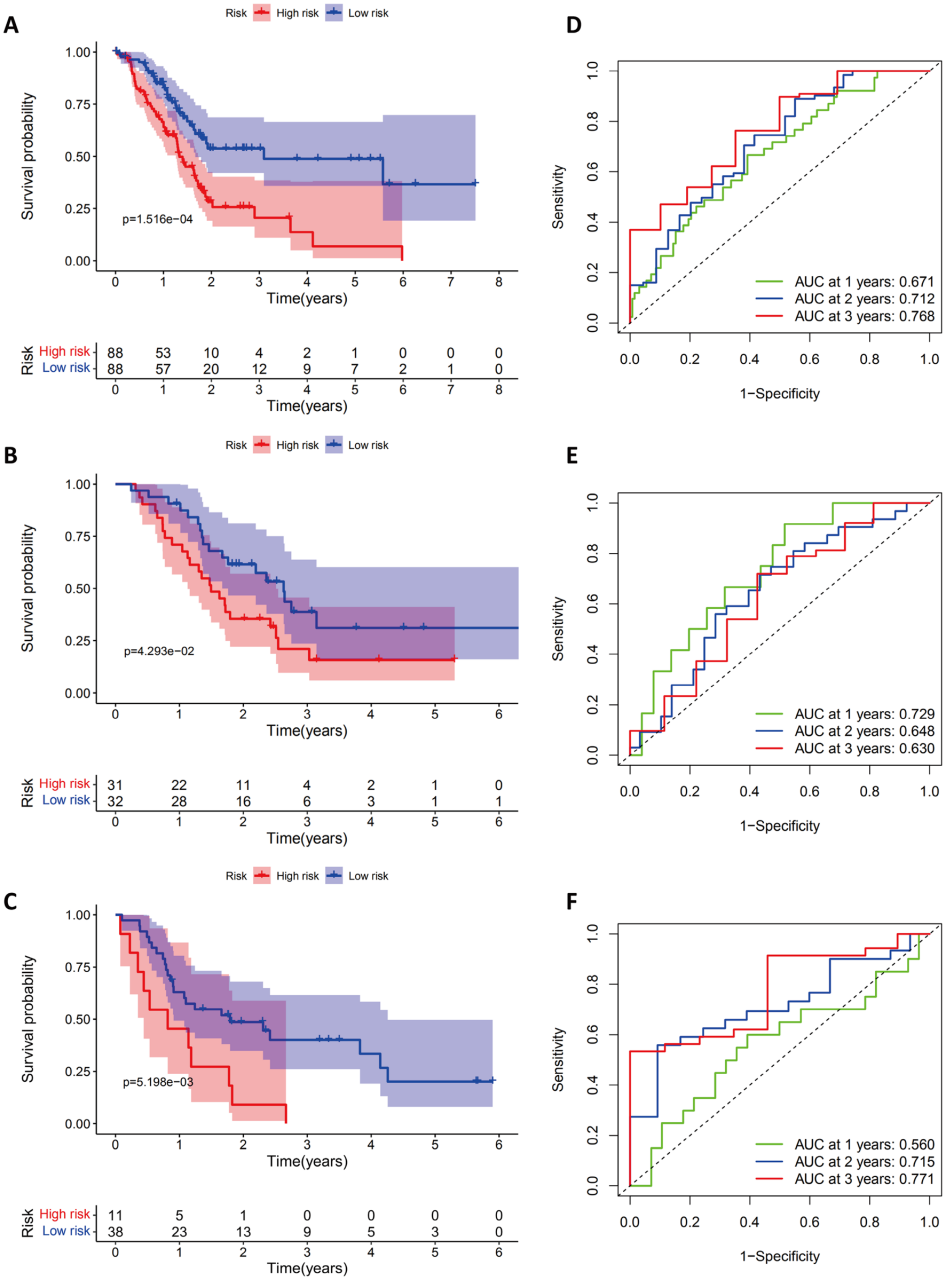
Survival Analysis of the PAAD Cohort. Kaplan–Meier analysis of PAAD patients in the TCGA (**A**), GSE57495 (**B**), and GSE78229 cohorts (**C**) by high-risk and low-risk groups. A comparison of the ROC curves for the risk scores of PAAD patients for the TCGA (**D**), GSE57495 (**E**), and GSE78229 cohorts (**F**).

The TCGA and GEO cohorts were analyzed for clinicopathologic characteristics. Kaplan–Meier analysis was performed on the survival curves of the low-risk and high-risk patient groups. In the high-risk group, the probability of survival after one year was significantly lower (*p* = 0.001 in TCGA, *p* = 0.043 in GSE57495, *p* = 0.005 in GSE78229, *p* = 0.017 in GDS4336, *p* = 0.031 in GSE85916, and *p* = 0.003 in ICGC-PACA-AU) (Fig. 4A–C and Supplementary Fig. 12). The areas under the receiver operating characteristic curves (AUCs) for 1-year, 2-year and 3-year OS were 0.671, 0.712 and 0.768 in the TCGA-PAAD dataset (Fig. 4D); 0.729, 0.648 and 0.630 in the GSE57495 dataset (Fig. 4E); and 0.560, 0.715, and 0.771 in the GSE78229 dataset (Fig. 4F).

### Prognosis value of the ARG-based risk model in the TCGA-PAAD dataset

**Figure 5:**
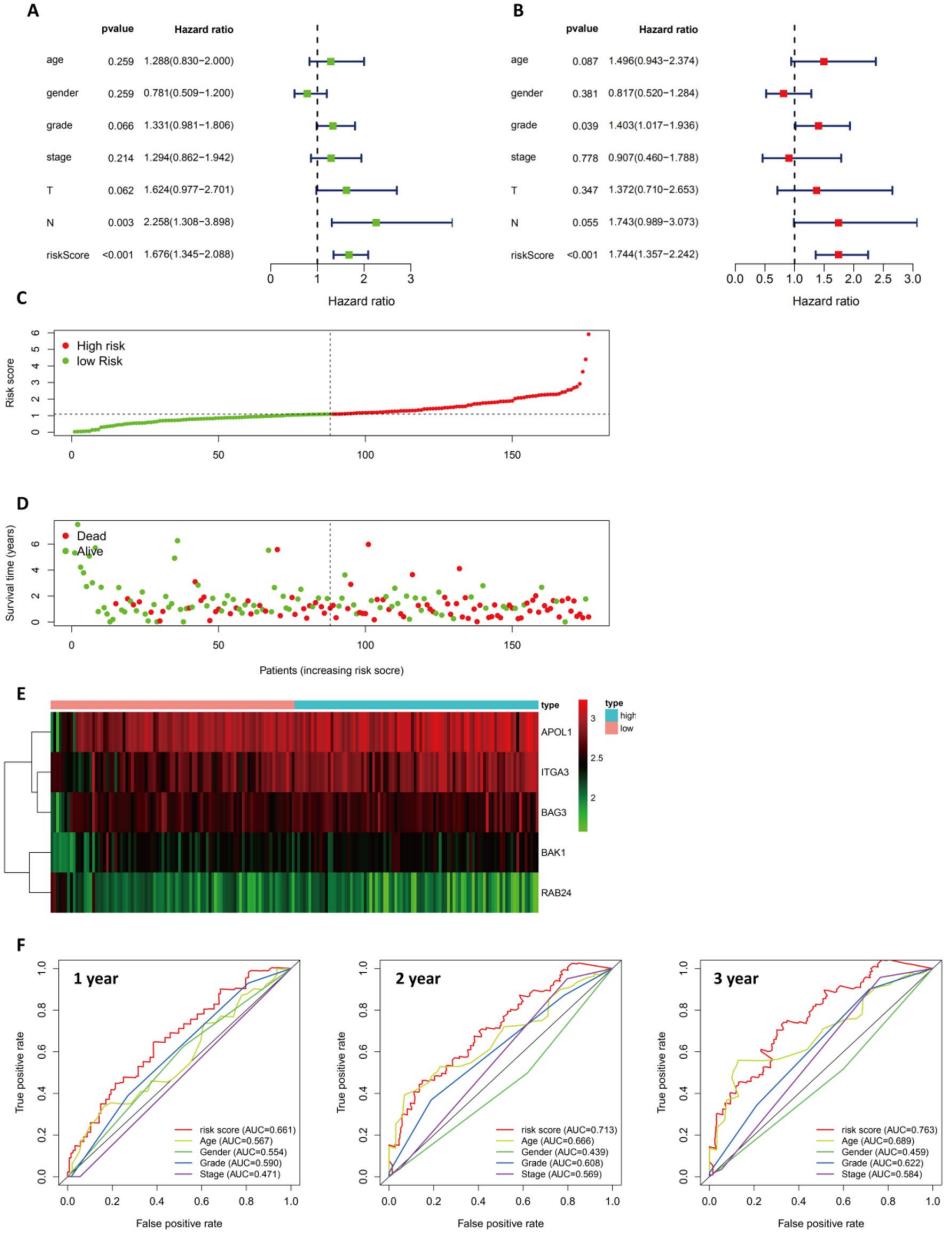
Examination of the Prognostic ARG Signature in the TCGA-PAAD Dataset. In PAAD, the forest plot of univariate and multivariate Cox regression analyses is shown in (**A**–**B**). (**C**) Prognostic index distribution. (**D**) Survival data for patients in the low- and high-risk groups. (**E**) An interactive heatmap of gene expression profiles for the included ARGs. (**F**) The AUC values of the risk score at 1, 2, and 3 years, as well as the clinicopathological characteristics.

We conducted univariate and multivariate analyses to identify factors that might be associated with a poor/better prognosis in pancreatic cancer patients registered in TCGA (Fig. 5A–B). Multivariate analysis confirmed the significance of the grade and risk score in the forest map. Thus, risk scores based on the five ARGs were independently associated with patient outcomes (HR = 1.744, 95% CI = 1.36–2.24).

In the next step, a ranking of the risk scores was used to analyze the distribution of risk scores and survival status (Fig. 5C–D). Based on the results of the study, patients with higher risk scores were more likely to die than those with lower risk scores. The differential expression profiles of the 5 risk score-associated ARGs between the low-risk group and the high-risk group are shown in the heatmap of Fig. 5E. As a means of further examining the diagnostic efficiency of the five risk score-associated ARGs and the clinical characteristics, 1-, 2-, and 3-year ROC curves have been generated for the risk score and the clinical characteristics, as shown in Fig. 5F.

### Investigation of the ARG-based risk model in the testing group

**Figure 6:**
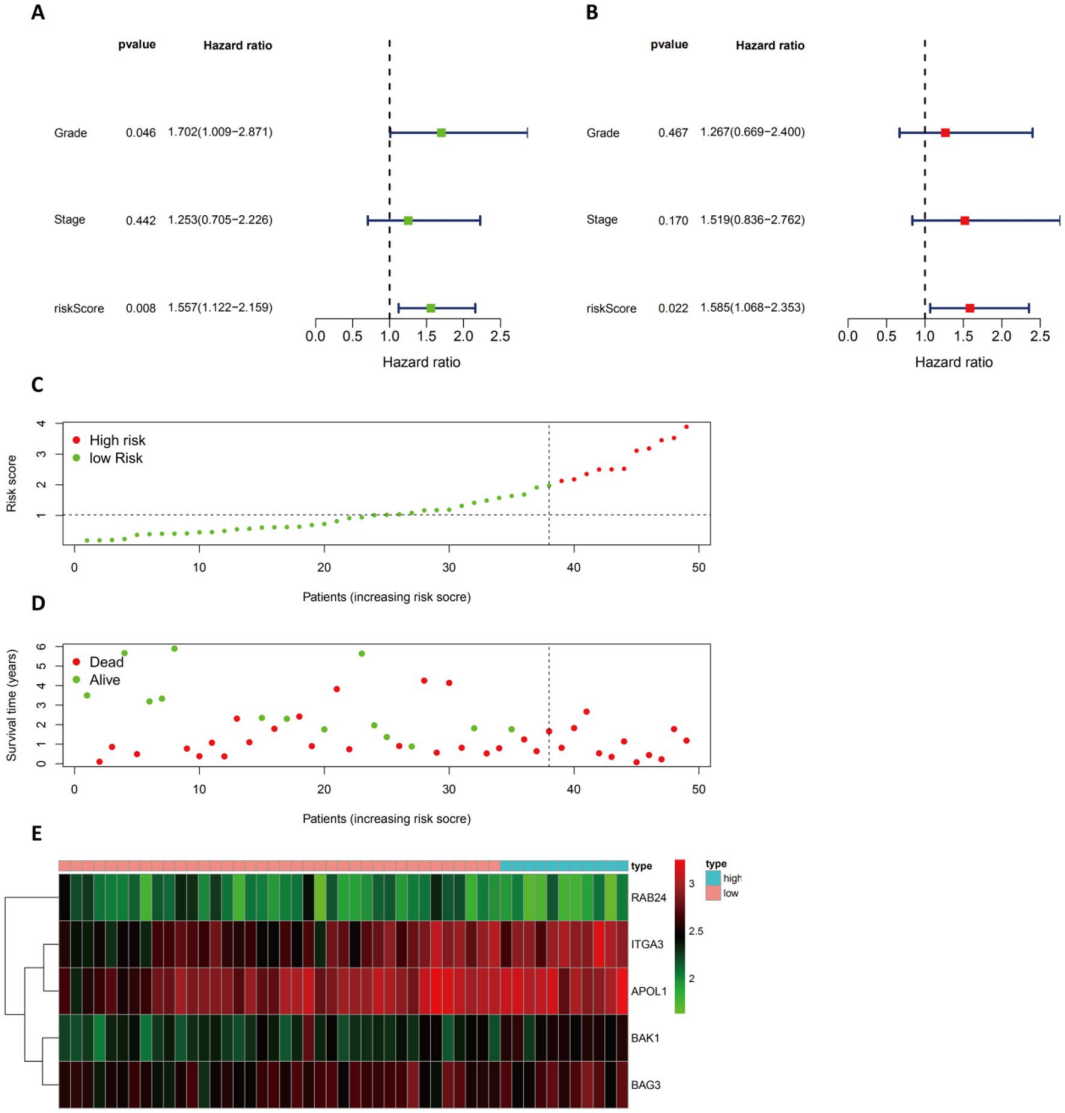
Evaluation of the Prognostic ARG Signature in the GSE78229 Dataset. (**A**–**B**) Forest plot illustrating the univariate and multivariate Cox regression analyses in PAAD. (**C**) Distribution of prognostic index. (**D**) Survival status of patients in the low- and high-risk groups. (**E**) The expression profile of the ARGs included in the heatmap.

Following the analysis of the data from the TCGA datasets of pancreatic cancer cohorts, univariate and multivariate Cox regression analyses were conducted to examine the impact of the ARG signature on the prognosis of pancreatic cancer patients in external datasets. In GSE78229, both univariate and multivariate Cox regression analyses revealed that the risk score remained significant after multivariate analysis (Fig. 6A–B). This finding indicates that the risk score is independently related to the prognosis of patients (HR of 1.585, 95% CI of 1.08–2.35). From the GSE78229 dataset, groups of high-risk and low-risk patients were ranked according to risk scores to analyze the distribution of risk scores and survival status and the expression profiles of risk-associated ARGs (Fig. 6C–E). Therefore, ARG-based models were confirmed to be accurate in the independent validation pancreatic cancer cohorts.

**Figure 7:**
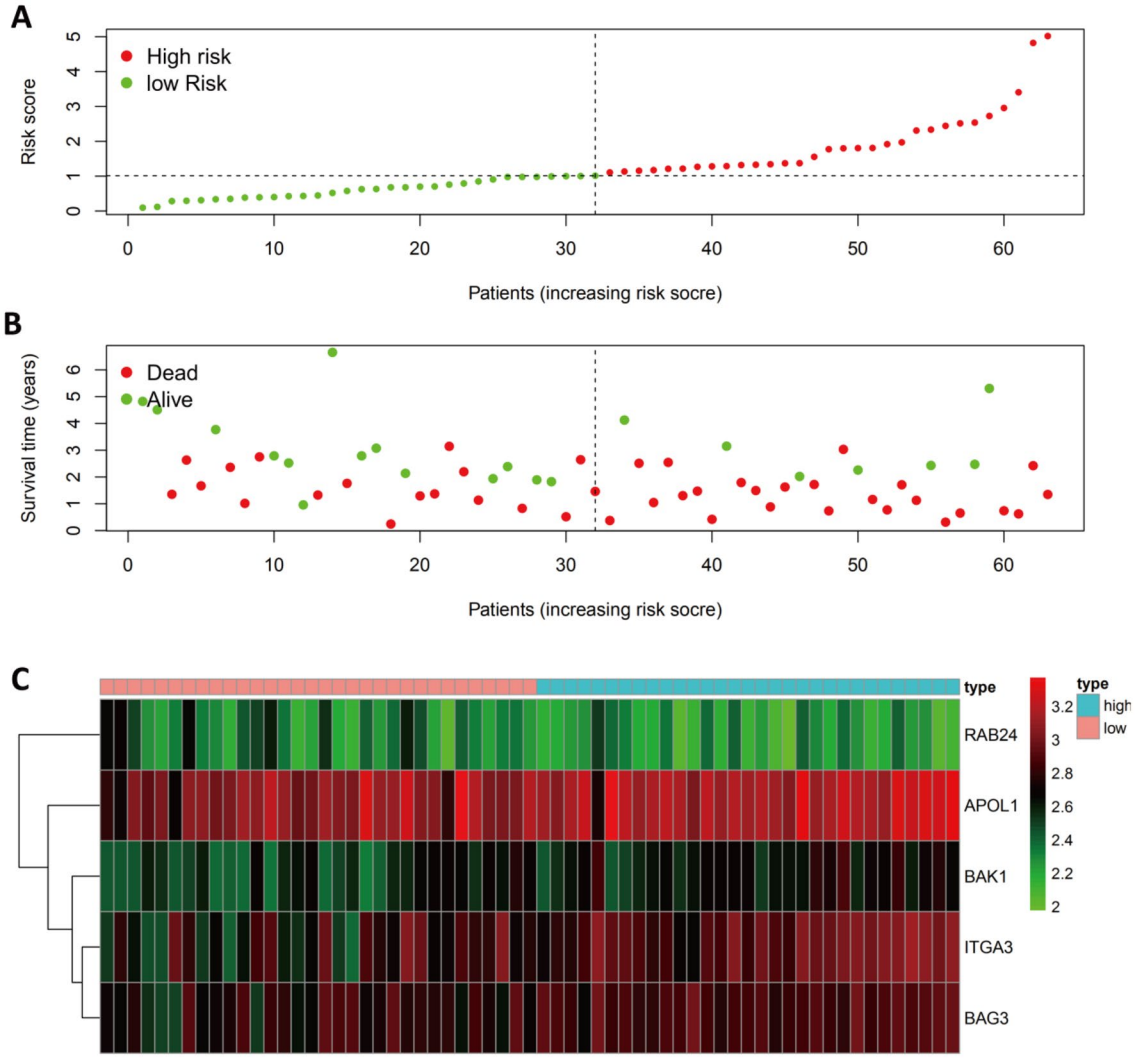
Verification of the Prognostic ARG Signature in the GSE57495 Dataset. (**A**) Graph illustrating the distribution of the prognostic index. (**B**) The survival status of patients in low-risk and high-risk groups. (**C**) A heatmap showing the expression profiles of included ARGs.

As the GSE57495 dataset does not contain any clinical information, it was not considered as part of the univariate or multivariate Cox regression analysis. However, we evaluated the distribution of risk scores and survival variables and the expression profiles of the risk-associated ARGs for the high-risk and low-risk pancreatic cancer patient groups based on their risk scores in the GSE57495 dataset (Fig. 7A–C). Supplementary Figs. 3–5 list the results from other external datasets (GDS4336, GSE85916, and ICGC-PACA-AU).

### Differences between high-risk and low-risk groups in terms of immune cell infiltration

**Figure 8:**
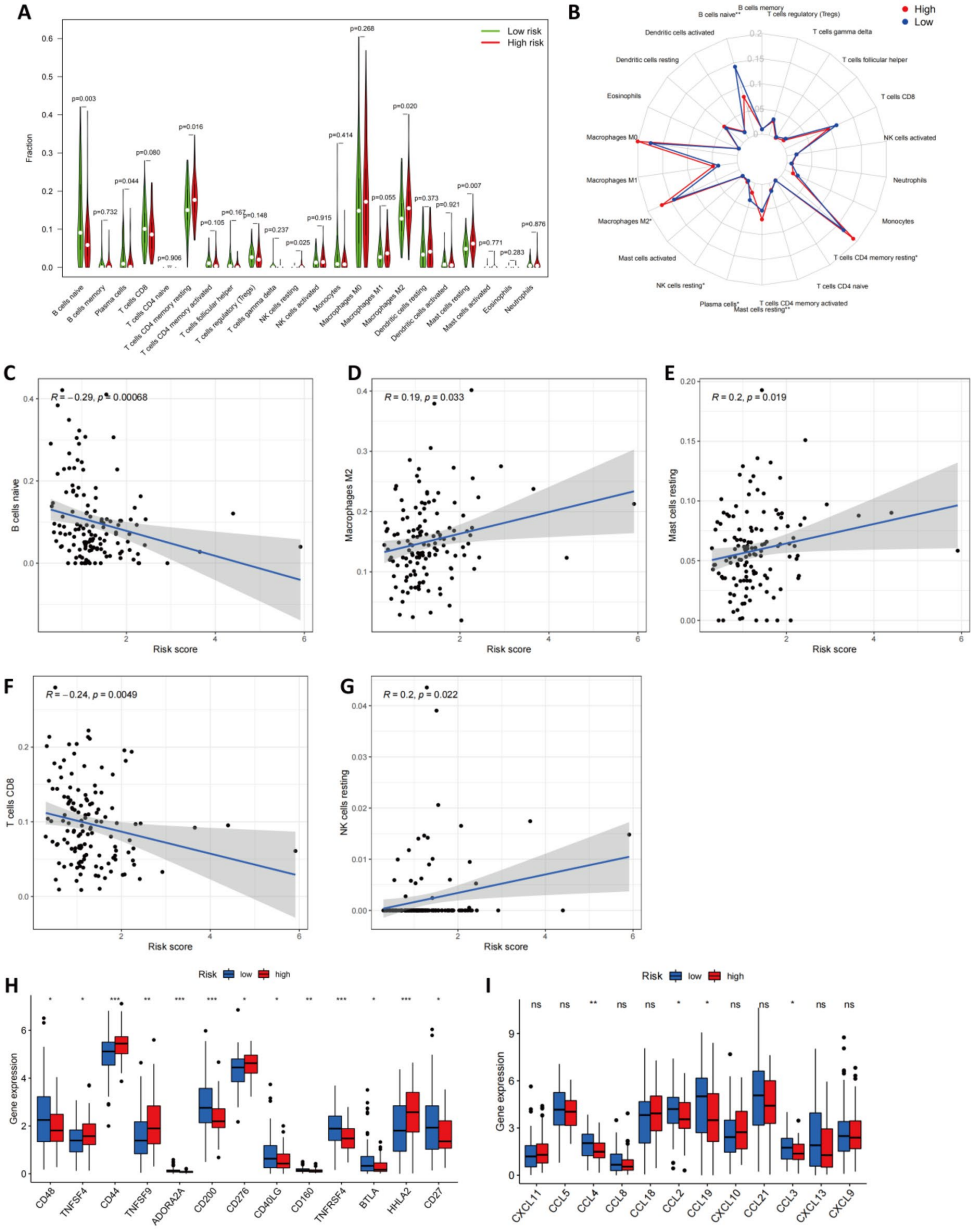
Correlation between the ARG-Based Model and the Infiltration of Immune Cells. The difference in the infiltration of immune cells between the high‐ and low‐risk groups are shown in (**A**) and summarized in (**B**). (**C**–**G**) An analysis of Spearman correlations of risk scores with immune cell infiltration levels was performed, and results with statistical differences are illustrated. (**H**) The level of checkpoint genes was determined in the low- and high-risk groups. (**I**) The expression of TLS-associated genes was determined in the low- and high-risk groups. An analysis of Spearman correlations of risk scores with immune cell infiltration levels was performed.

In our next analysis, we examined how ARGs impacted the prognosis of patients with pancreatic cancer. As autophagy is correlated with immune cell infiltration and immune cell infiltration has been linked to cancer development and prognosis, we used CIBERSORT to compare the abundance of 22 immune cell types between the high- and low-risk groups in TCGA-PAAD (Fig. 8A–B). A total of six immune cells were found to show varying degrees of infiltration between the two groups with respect to the 22 types that were studied: the infiltration levels of naive B cells (*p* = 0.003) and plasma cells (*p* = 0.044) were higher in the low-risk group, and the infiltration levels of memory CD4 T cells (*p* = 0.016), resting NK cells (*p* = 0.025), M2 macrophages (*p* = 0.020) and mast cells (*p* = 0.007) were higher in the high-risk group. In addition, the CD8 T-cell infiltration level was nearly significantly lower in the high-risk group (*p* = 0.080). The immune infiltration landscape is summarized in a radar plot in Fig. 8B. Additionally, the correlation between the risk score and the infiltration of the seven immune cells was investigated by Spearman correlation analysis. According to our results, the infiltration levels of naive B cells (r = −0.29, *p* < 0.001, Fig. 8C), M2 macrophages (r = 0.19, *p* = 0.033, Fig. 8D), mast cells (r = 0.20, *p* = 0.019, Fig. 8E), CD8 T cells (r = -0.24, *p* < 0.001, Fig. 8F) and NK resting cells (r = 0.20, *p* = 0.022, Fig. 8G) were statistically correlated with the ARG risk scores.

Multiple immunotherapies produce therapeutic effects by expressing immune checkpoint genes. Therefore, we next compared the expression of checkpoint genes between the two groups (Fig. 8H). It was statistically significant that there was a difference between the two groups in terms of all the checkpoint genes. The expression of CD48, ADORA2A, CD200, CD40LG, CD160, TNFRSF4, BTLA, and CD27 was obviously lower in the high-risk group, and that of TNFSF4, CD44, TNFSF9, CD276, and HHLA2 was higher in the high-risk group than in the low-risk group. In addition, we investigated the level of tumor tertiary lymphoid structure (TLS)-related genes between the low/high-risk group according to previous reports^26^, and the results indicated that the level of CCL2, CCL3, CCL4, as well as CCL19 was lower in the high-risk group than in the low-risk group (Fig. 8I).

### Verification of ARG expression in tumor samples and cell lines

**Figure 9:**
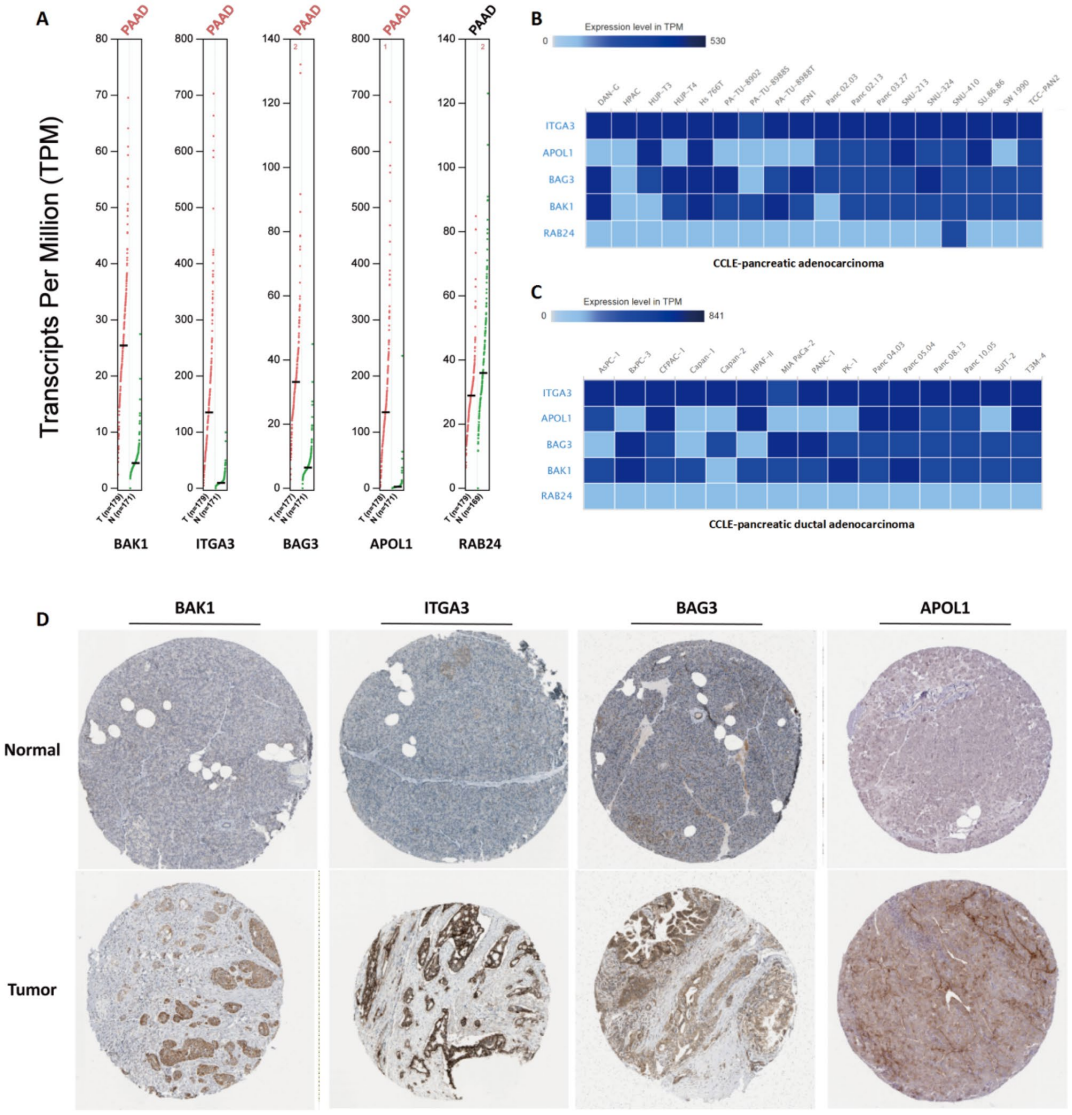
Differential Expression of the 5 Signature ARGs in PAAD In Vitro and In Vivo. (**A**) ARG expression in pancreatic cancer tumor tissues and normal tissues according to the TCGA database. (**B**–**C**) The level of BAK1, ITGA3, BAG3 and APOL1 in most pancreatic cancer cell lines and pancreatic ductal adenocarcinoma cell lines based on the CCLE database. (**D**) Immunohistochemical staining of BAK1, ITGA3, BAG3 and APOL1 in pancreatic cancer tumor tissues and normal tissues. These data were obtained from the HPA database.

We collected data from pancreatic cancer patients and cell lines to verify the expression of ARGs. GEPIA was used to investigate clinical sequencing information from the TCGA and GTEx databases. The expression levels of BAK1, ITGA3, BAG3, and APOL1 were statistically increased in pancreatic cancer samples versus normal samples, while those of RAB24 was decreased (Fig. 9A). These results were confirmed with the CCLE database. BAK1, ITGA3, BAG3 and APOL1 were highly expressed in most pancreatic cancer and pancreatic ductal adenocarcinoma cell lines (Fig. 9B–C), while RAB24 was poorly expressed.

Using the HPA database, we conducted a further analysis of ARG expression. Compared with those from the normal controls, immunohistochemical results from the pancreatic tissues revealed statistical increases in the expression of BAK1, ITGA3, BAG3, and APOL1 in pancreatic cancer patients; no immunohistochemical data were available for RAB24 (Fig. 9D).

### Functional enrichment analysis and small molecule drug screening using DEGs

**Figure 10:**
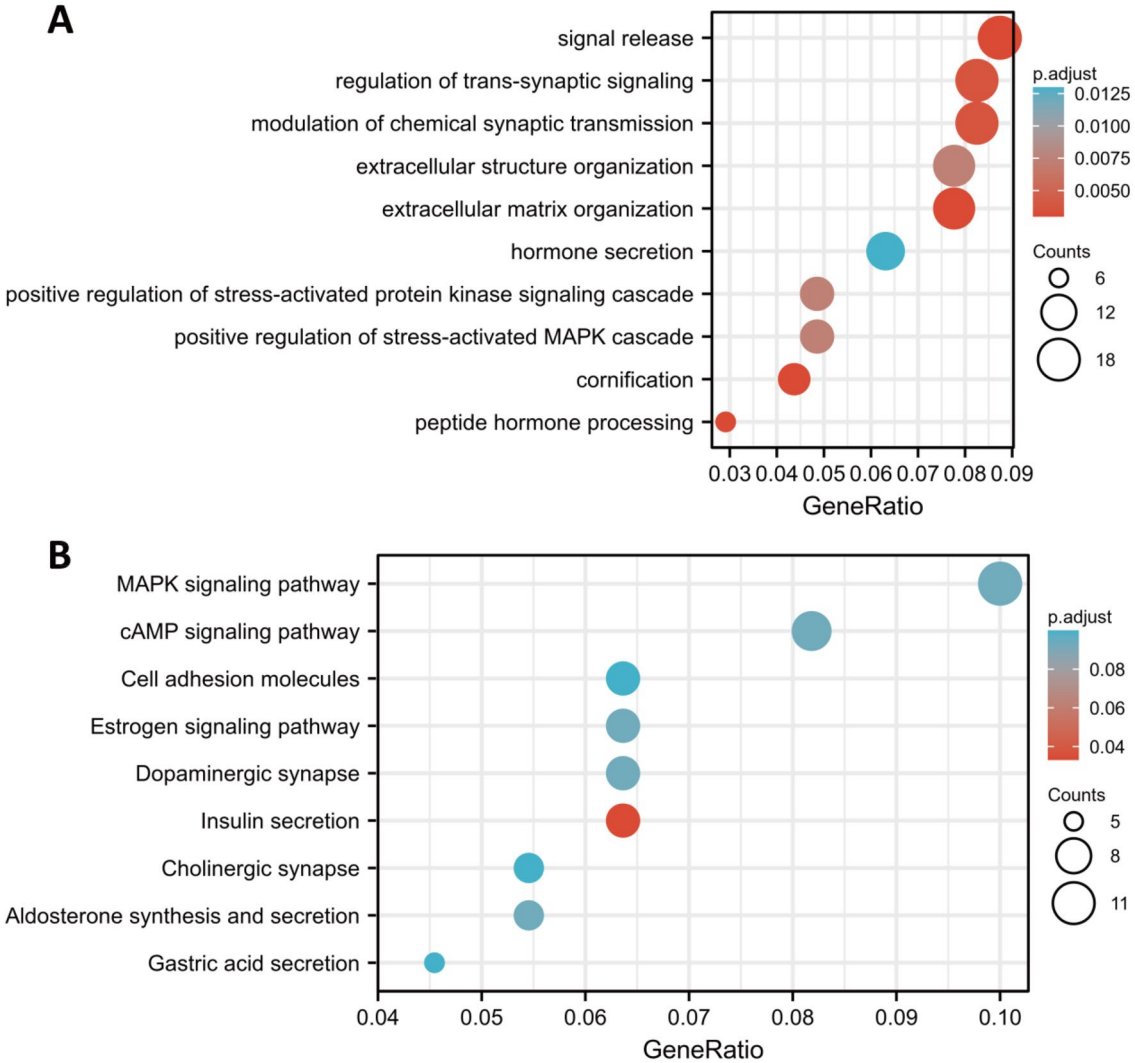
An analysis of functional enrichment between the low- and high-risk groups (**A**) GO-BP analysis and (**B**) KEGG pathway analysis.

Further GO-BP and KEGG analyses were conducted on DEGs between the high-risk and low-risk groups. According to our analysis, the top three BPs pathways were signal release, transsynaptic signaling, and chemical synaptic transmission (Fig. 10A), and the top three KEGG pathways were MAPK, cAMP, and cell adhesion molecules (Fig. 10B).

**Figure 11:**
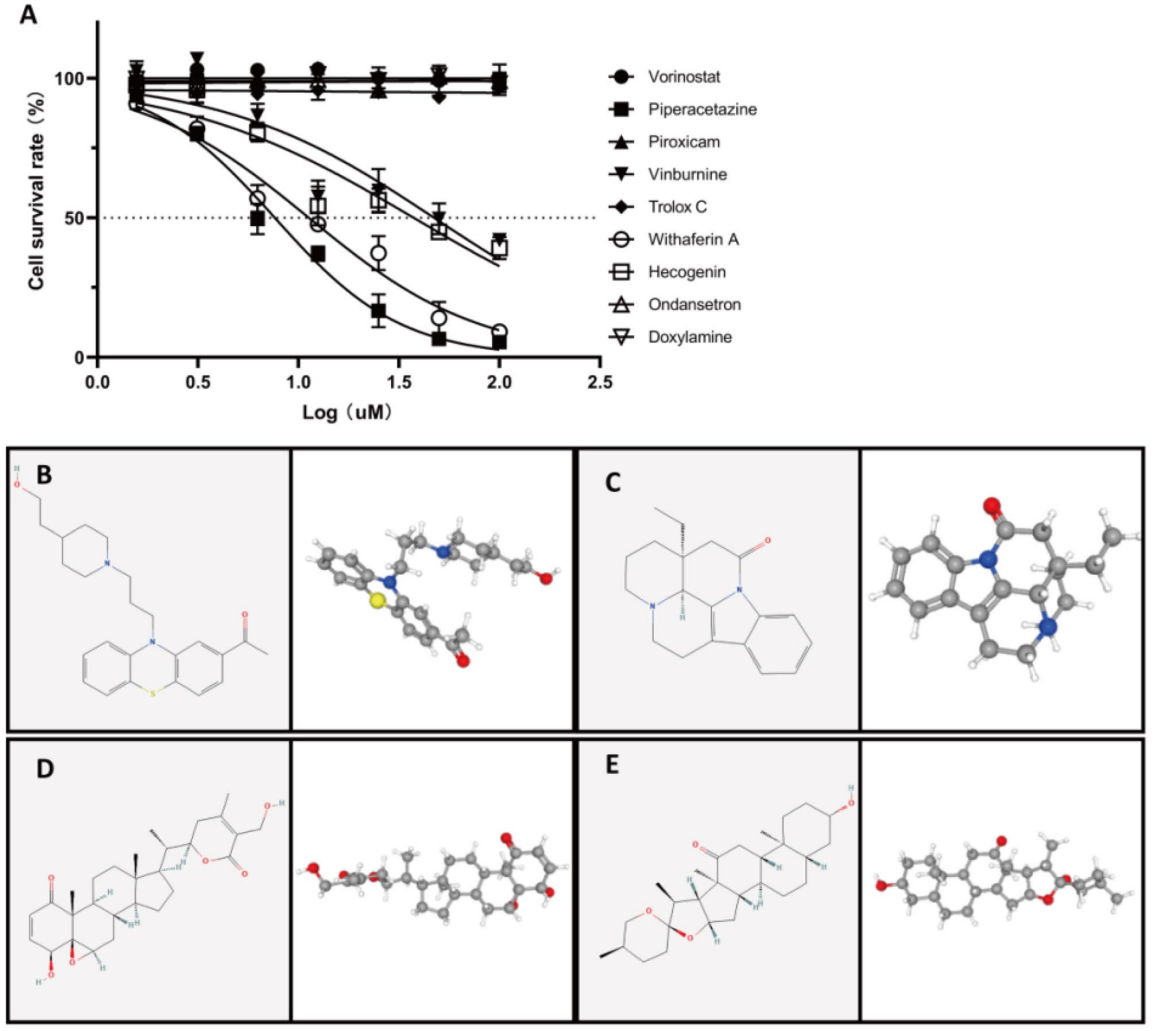
Examination of Potential Small-Molecule Compounds. CMap has been used to screen for small-molecule compounds based on DEGs uploaded to the site. The IC50 values of the drugs were further examined in PANC-1 cells (**A**). Furthermore, for each of the four compounds that had been screened, the PubChem website was used to visualize the results. The 2/3D spatial structure of piperazine (**B**), vinburnine (**C**), withaferin A (C), and hecogenin (**D**).

Our research matched upregulated and downregulated DEGs with small-molecule therapies using the Connectivity Map (CMap) website to identify potential drugs for PAAD. Table 2 lists the 9 most significant small-molecule drugs and their similarity scores. The IC50 values of the drugs were further examined in PANC-1 cells. We identified 4 drugs, namely, piperacetazine (IC50 = 7.627 μM), vinburnine (IC50 = 47.28 μM), withaferin A (IC50 = 11.26 μM), and hecogenin (IC50 = 37.45 μM) as potential drugs for improving the prognosis of patients with pancreatic cancer (Fig. 11A). Utilizing the PubChem website, their 2/3D spatial structure were visualized (Fig. 11B–E). These potent small-molecule drugs could reverse autophagy-induced gene expression, which provides a framework for developing targeted drugs for the treatment of PAAD. There are still many more studies to be conducted to investigate the usefulness of these candidate drugs for PAAD treatment.

**Table 2:** The Screened Drugs for PAAD Treatment by CMAP.

Rank	cmap name	Mean	n	Enrichment	*p*	Specificity
1	Vorinostat	−0.458	12	−0.534	0.00108	0.3274
2	Piperacetazine	−0.336	4	−0.77	0.00567	0.0146
3	Piroxicam	−0.581	4	−0.71	0.01438	0.0292
4	Vinburnine	−0.372	4	−0.705	0.01566	0.0213
5	Trolox C	−0.454	4	−0.669	0.02697	0.0159
6	Withaferin A	−0.418	4	−0.661	0.02972	0.1475
7	Hecogenin	−0.319	4	−0.637	0.04201	0.0939
8	Ondansetron	−0.1	4	−0.627	0.0476	0.1053
9	Doxylamine	−0.243	5	−0.532	0.07332	0.2032

## Discussion

Pancreatic cancer is an aggressive malignancy with a low prognosis; the survival rate for patients suffering from pancreatic cancer is less than 10%^1^. Even though rapid advances have been made in the diagnostic and therapeutic treatment of malignant tumors, a lack of progress has actually been made for PAAD. According to a recent study, ARGs play an important role in PAAD^25^; however, less external datasets were used to verify these results. It is especially pertinent for PAAD, since sample sizes in each database are relatively small^20^. In this study, we evaluated the performance of an ARG-based prediction model in multiple datasets (GSE57495, GSE78229, GDS4336, GSE85916, and ICGC-PACA-AU). Considering the significance of autophagy in PAAD, in this study, we systematically constructed an ARG-based signature for PAAD to identify potential biomarkers for diagnosis and treatment.

First, we identified differentially expressed ARGs by combining the GTEx and TCGA datasets due to a lack of control samples in the TCGA database; then, we utilized GO-BP analysis and KEGG analysis to verify the role of ARGs in PAAD. ARG-based models were further established by univariate and multivariate Cox analyses. A final risk score was calculated as [Expression level of BAK1 × (0.34085)] + [Expression level of ITGA3 × (0.38309)] + [Expression level of BAG3 × (0.34635)] + [Expression level of APOL1 × (0.25892)] + [Expression level of RAB24 × (−0.67519)]. Evidence from the TCGA cohort and multiple external data sets indicate that ARGs play an important role in the prognosis of PAAD patients, even though the size of each cohort is relatively small. Overall, the ARG-based model in this study exhibits good universality and prognostic value.

In multivariate Cox analysis of the five ARGs, only ITGA3 (HR = 1.47, 95% CI = 1.13–1.99) and RAB24 (HR = 0.51, 95% CI = 0.35–0.74) were identified as independent ARGs. Integrin alpha 3 (ITGA3) belongs to the family of integrins, which are heterodimeric integral membrane proteins that function as cell surface adhesion molecules. This gene has been reported in several autophagy-related prognostic models for various tumors, indicating its important role in the progression of tumors^27–32^. Mechanistically, it has been reported that miRNA-524-5p inhibits the progression of papillary thyroid carcinoma cells by targeting FOXE1 and ITGA3 in cell autophagy and cycling pathways^33^. In addition, the IκB kinase complex (IKK) triggers detachment-induced autophagy in mammary epithelial cells, resulting from decreased ITGA3-ITGB1 function, and these phenomena are associated with cancer progression and metastasis^34^. However, the related literature is still limited, and the role of ITGA3 should be further studied. As the only protective factor, high RAB24 expression was associated with a good prognosis. RAB24 is a small GTPase belonging to the Rab subfamily of Ras-related proteins; it regulates intracellular protein trafficking and has been shown to be important in autophagosome maturation^35–39^. RAB24 was the first protein to be shown to be necessary in the very late stages of basal autophagy^40^ and has been linked to various diseases, including carotid atherosclerosis^41^ and multiple sclerosis^42^. In studies in tumors, RAB24 was identified as a direct target of miR-615-5p in hepatocellular carcinoma (HCC). Research has shown that the downregulation of miR-615-5p expression and the upregulation of RAB24 expression promotes epithelial-mesenchymal transition, adhesion and vasculogenic mimicry in HCC cells, which enhance metastasis^43^. This finding is somewhat similar to that in a study of an ARG-based model for prostate cancer^44^, which also identified RAB24 as a protective factor. One possible explanation is that changes in RAB24 expression are a result of autophagy-associated physiological phenomena rather than a cause of tumorigenesis.

Although BAK1, BAG3 and APOL1 were not identified as independent risk factors, they are still underlying biomarkers and targets in PAAD treatment because the small sample size reduced the statistical power. BAK1 (known as BCL2 antagonist/killer 1) is an inhibitor of the BCL2 protein, which is the central player in the mitochondrion-dependent apoptotic program. As a result of multiple apoptotic events, mitochondrial outer membrane permeabilization (MOMP) has been shown to result in the release of cytochrome c into the cytosol to activate caspases, and BAK is a key effector involved in MOMP. However, BAK1 is also reported to be involved in autophagy^45^. Beclin-1 is an important regulatory hub to which proautophagic and antiautophagic proteins can bind. Bcl-2 family members are widely reported to modulate Beclin-1-dependent autophagy, which can be inhibited by BAK1^46–49^. Further, BCL2 and BCL2L1/BCL-XL are thought to inhibit autophagy indirectly through an interaction with the proapoptotic members of the BCL2 family, BAX and BAK1^50,51^. It is known as BCL2-associated athanogene 3 (BAG3), which is a multifunctional HSP70 cochaperone and antiapoptotic protein that interacts with the ATPase domain of HSP70 through its C-terminal BAG domain, and plays a crucial role in maintaining cellular proteostasis^52^. Along with HSP70 and LC3, BAG3 can also target polyubiquitinated client proteins for degradation by autophagy. BAG3 therefore plays a key physiological role in the regulation of both proteasomal degradation and autophagy, which are major cellular pathways for protein degradation^53–55^. Aberrant expression of BAG3 has been linked to different cancer entities^56–61^. Due to its ability to promote cell survival signaling by interacting with distinct client proteins in complex with HSP70, BAG3 overexpression contributes to the development of apoptosis resistance in various types of tumors^62^. APOL1 (apolipoprotein L 1) is a secreted high-density lipoprotein^63^, which has been demonstrated to participate in the progression of hyperlipidemia, obesity and atherosclerosis^64–67^. APOL1 overexpression induces autophagy and autophagy-associated cell death in a variety of cancer cell types^68,69^.

Understanding the cellular and molecular mechanisms underlying autophagy-related immune modulation is a prerequisite for the development of immunotherapy-based targeted approaches for this deadly malignancy. Via immune infiltration analysis, we found that the high-risk group exhibited increased levels of memory CD4 T cells, NK cells, M2 macrophages, and mast cell infiltration. Conversely, this group exhibited lower levels of naive B-cell and plasma cell infiltration than the low-risk group. The infiltration of another CD8 T-cell population was almost significantly decreased in the high-risk group compared with the low-risk group (*p* = 0.08). Tertiary lymphoid structures (TLSs) are composed of complex aggregates of cytotoxic lymphocytes, B lymphocytes (including plasma cells) and dendritic cells^70^. For patients with solid tumors, the presence of TLSs has been associated with a favorable outcome^71^. Studies have shown that cytotoxic CD8^+^ T cells are important effector cells that contribute to adaptive immunity by specifically recognizing and wiping out tumor cells, and they are thereby associated with improved survival in cancer patients^20,72^. In previous studies, it was found that infiltration of CD20^+^ B cells in ovarian, non-small lung and cervical cancers was associated with improved survival and decreased relapse rates^20,72,73^. According to two recent prognostic models of PAAD, low numbers of B cells (plasma cells) and CD8^+^ T cells were associated with a poor prognosis in the disease^20,74,75^. Monocytes can differentiate into M1 and M2 macrophages, and M2 macrophages exhibit immunosuppressive and tumor-promoting roles^76^. Therefore, high M2 macrophage infiltration levels may limit the antitumor response. In terms of CD4 + memory T cells, two recent studies reported that a high level of CD4 + naive/CD4 + memory cell infiltration predicted improved OS in PAAD and non-small-cell lung cancer^77,78^, which partly supports our results. In conclusion, the overall effect of the ARGs in this signature on the immune microenvironment of PAAD involves a comprehensive and complex process. We hypothesized that the risk score ultimately reflects the degree of antitumor response suppression because the high-risk group had worse survival outcomes than the low-risk group in multiple databases. It should be noted that the investigation of the expression of checkpoint genes and TLS-associated genes also revealed obvious differences between the two groups, indicating that the ARG-based model may provide potential targets for PAAD treatments, which is very similar to the results of another research model we built^20^. Interestingly, several checkpoints were more highly expressed in the high-risk group, indicating a worse prognosis in the PAAD cohort. Among the genes, several genes (CD44^79^, TNFSF9^80^, and CD276^81^) have been reported and demonstrated previously; however, the high expression of HHLA2 is inconsistent with the results in a newly published study^82^. Exploration of the phenomena and mechanisms may require more experiments.

The DEGs were then analyzed for GO-BP and KEGG enrichment. A GO analysis revealed that signal release, regulation of transsynaptic signaling, and modulation of chemical synaptic transmission, all of which were associated with hormone secretion. Other evidence from both CMap (Table 2) and IC_50_ (Fig. 11A) examination showed that piperacetazine serves as a dopamine receptor antagonist and has the potential to be used to treat PAAD. Recently, the dopamine D2 receptor (D2R) family was demonstrated to be upregulated in many cancers and tied to stemness^83^. Interestingly, the expression of D2R and its associated G protein Gai2 has been reported to be obviously upregulated in pancreatic ductal adenocarcinoma tissue samples^84^. In addition, there is increasing evidence that autophagy is closely related to the activity of dopamine receptors^85^. Whether autophagy can regulate the activity of dopamine receptors and affect the occurrence and development of pancreatic cancer must be explored. Comparing the high-risk and low-risk groups, KEGG analysis revealed that DEGs were mainly enriched in MAPK and cAMP pathways, which are crucial for the activation and regulation of autophagy^86–89^. Interestingly, potential treatments targeting autophagy via MAPK^90,91^ and cAMP^92^ in PAAD have been recently reported. Further investigation was conducted on potential small-molecule drugs with significant negative fractions. Although several of these drugs are not clinically used, comparing the functions of different drugs targeting differentially expressed ARGs may provide putative biomarkers for further validation as changes in gene expression in cancer can influence treatment outcomes^20,93^. In our analysis, the DEGs were uploaded to the CMap website, and the IC50 values of the predicted drugs were further examined in PANC-1 cells. In addition to piperacetazine described above (IC50 = 7.627 μM), withaferin A also showed a strong killing effect on pancreatic cancer cells (IC50 = 11.26 μM). According to research, withaferin A, a natural compound derived from the ashwagandha plant Withania somnifera^94^, has anti-diabetic properties^95^, protects the liver from injury caused by acetaminophen^96^, and triggers apoptosis in various cancers, including breast^97^, prostate^98^, colorectal^99^, non-small-cell lung^100^ and pancreatic cancer. In addition, withaferin A also induces incomplete autophagy by suppressing the fusion of autophagosomes and lysosomes in human pancreatic cancer cells^101^.

To conclude, the identified risk-associated ARGs may provide a basis for developing PAAD treatments involving autophagy. Importantly, the predictive value of the ARGs-based signature was confirmed by external cohort of PAAD, indicating that this model is able to benefit formulation of precise treatment plans. In spite of this, additional prospective experiments are necessary to determine the clinical relevance of this model in defining the optimal personalized targeted treatments and to explore treatments that target ARGs in PAAD^20^.

## Methods

The authors declare that all the methods in this article were performed in accordance with the relevant guidelines and regulations in the editorial and publishing policies of Scientific Reports (https://www.nature.com/srep/journal-policies/ editorial-policies#experimental-subjects).

### Study design and data collection

Figure 1 illustrates the experimental design and data analysis flow. Transcription profiles and clinical data of TCGA-PAAD patients and normal controls from the GTEx database were obtained from the UCSC Xena website (http://xenabrowser.net/datapages/), and these data were used as a training group. Microarray datasets (GSE57495, GSE78229, GDS4336, and GSE85916) were downloaded from the Gene Expression Omnibus (GEO) portal (https://www.ncbi.nlm.nih.gov/geo/). RNA-seq data from the PACA-AU cohort were downloaded from the ICGC Data Portal (https://dcc.icgc.org/) for further validation of the signature and used as an external test group. The ARGs were obtained from the Human Autophagy Database (HADb) (http://www.autophagy.lu/clustering/) (listed in Supplementary Table 3).

### Identification of DEGs and extraction of ARGs

The normalized gene expression was calculated using fragments per kilobase of transcript per million mapped reads (FPKM) and log2-based transformation. Following this, the "sva" package of R was used to normalize RNA expression profiles and to remove batch effects. Using the limma package, we identified the DEGs with |logFC|> 1.5 and adjusted *p* < 0.01 by the limma package. We then extracted the ARGs from the combined PAAD cohort data.

### Construction of the prognostic risk model

Utilizing the TCGA-PAAD database, a univariate Cox regression analysis was conducted to identify differentially expressed ARGs with prognostic significance. To construct a potential independent prognostic ARG model for pancreatic cancer, the identified prognostic ARGs were further included in a multivariate Cox regression calculation. After establishing a formula for the risk score, we calculated the risk score in each case as follows:

$$ {\text{RiskScore}} = \sum\nolimits_{t = 1}^{n} {{\text{Coef}}_{{\text{i}}} \times {\text{X}}_{{\text{i}}} } $$

Coefi represents the correlation coefficient of each ARG and X represents gene expression. Using the median risk score of the TCGA-PAAD cohort as a cutoff value, the external datasets patients were divided into high- and low-risk groups in accordance with the cutoff value.

### Evaluation of the prognostic capacity of the ARG model

Both the TCGA cohort and external datasets (GSE57495, GSE78229, GDS4336, GSE85916 and ICGC-PACA-AU) were analyzed using R software. A Kaplan–Meier analysis was performed on the survival data based on ARGs for pancreatic cancer patients. Univariate and multivariate Cox regression analyses were used to identify the independent risk factors. The receiver operating characteristic (ROC) curves were generated by the timeROC package. The prognostic efficiency of the model was measured by the area under the ROC curve (AUC).

### Estimation of immune cell infiltration

The levels of 22 cancer-infiltrating immune cell subgroups were quantified using CIBERSORT to evaluate immune cell infiltration. Using Spearman correlation analysis, further relationships were explored between immunocellular subgroups infiltrating tumors and ARG expression. The expression of potential immune checkpoint genes^102–111^ and TLS-related genes^26^ was also investigated according to previous literature.

### Functional and pathway enrichment analysis

The DEGs between groups were analyzed with the “limma” and “clusterProfiler” packages to perform Gene Ontology biological process (GO-BP) analysis and Kyoto Encyclopedia of Genes and Genomes (KEGG)^112–114^ pathway enrichment analysis, as described previously^20,115^.

### Verification of ARG expression

To verify the expression of ARGs, the expression in cells from the Cancer Cell Line Encyclopedia (CCLE) database was visualized by Expression Atlas (https://www.ebi.ac.uk/gxa/home), and immunohistochemistry data for clinical samples were obtained from the Human Protein Atlas (HPA) website (https://www.proteinatlas.org/). The baseline immunohistochemical image data are provided in Supplementary Fig. 6.

### Identification of potential compounds

DEGs based on the ARG signature were divided into up- and downregulated gene groups and uploaded to the Connectivity Map website (https://portals.broadinstitute.org/cmap/), as our described previously^116^.

### CCK-8 assay and IC50 examination

The cytotoxicity of the predicted drugs against pancreatic cancer cells (PANC-1) was assessed by using a Cell Counting Kit-8. All cells were seeded in 96-well plates at a density of 5 × 103 cells/well at 37 °C. Each well was treated with different drug concentrations (100/50/25/12.5/6.25/3.125/1.5625 nM) or 1% DMSO for 48 h. Then, 10 µL of CCK-8 solution was added, and the cells were incubated for another 1 h. A microplate reader was used to determine absorbance at 450 nm and 650 nm (SpectraMax M5, Molecular Devices, USA). The assay was performed in triplicate. The IC50 was determined according to the previous literature. The 2D/3D structures of the screened drugs were further investigated by the PubChem website (pubchem.ncbi.nlm.nih.gov).

### Statistical analysis

Data were analyzed by R software version 4.0.4. Data following a normal or nonnormal distribution were compared using unpaired Student's t test and the Wilcoxon test, respectively, and the statistical significance threshold was set at *p* < 0.05^116^.

## Supplementary Information

Supplementary Information 1.

Supplementary Information 2.

Supplementary Information 3.

Supplementary Information 4.

## Data Availability

The datasets analyzed during the current research are all available in The Cancer Genome Atlas (http://cancergenome.nih.gov) repositories, Gene Expression Omnibus (http://www.ncbi.nlm.nih.gov/geo/), and International Cancer Genome Consortium Pancreatic Cancer Australian [ICGC-PACA-AU]) (https://dcc.icgc.org/). The data used to support the findings of this study are available from the corresponding author upon request.
