# Supplementary material for: Identification of an autophagy-related gene signature for predicting prognosis and immune activity in pancreatic adenocarcinoma
Source: Sci Rep. 2022 Apr 29;12:7006. doi: 10.1038/s41598-022-11050-w (PMC9054801; doi:10.1038/s41598-022-11050-w)

**A**


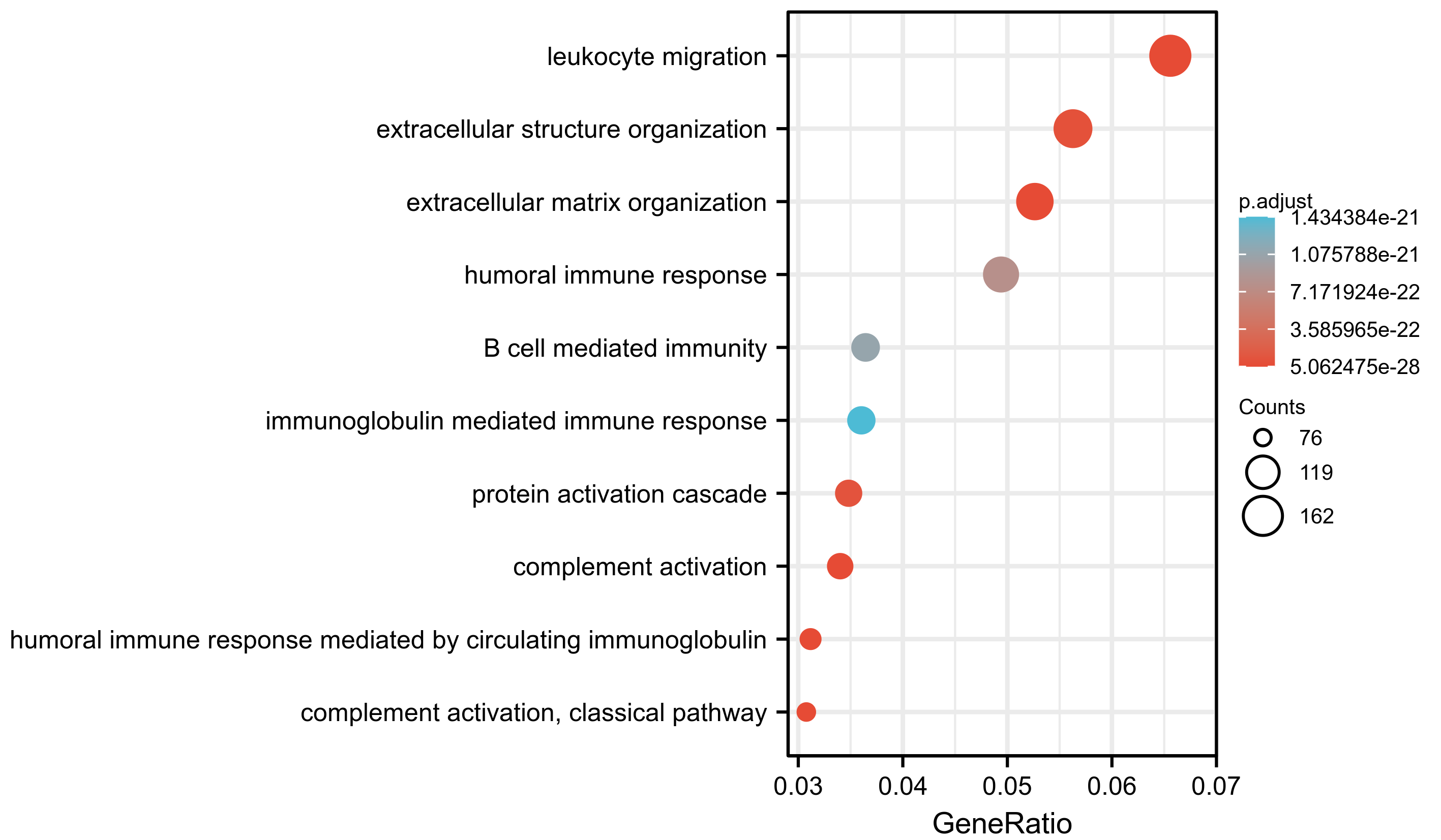


**B**


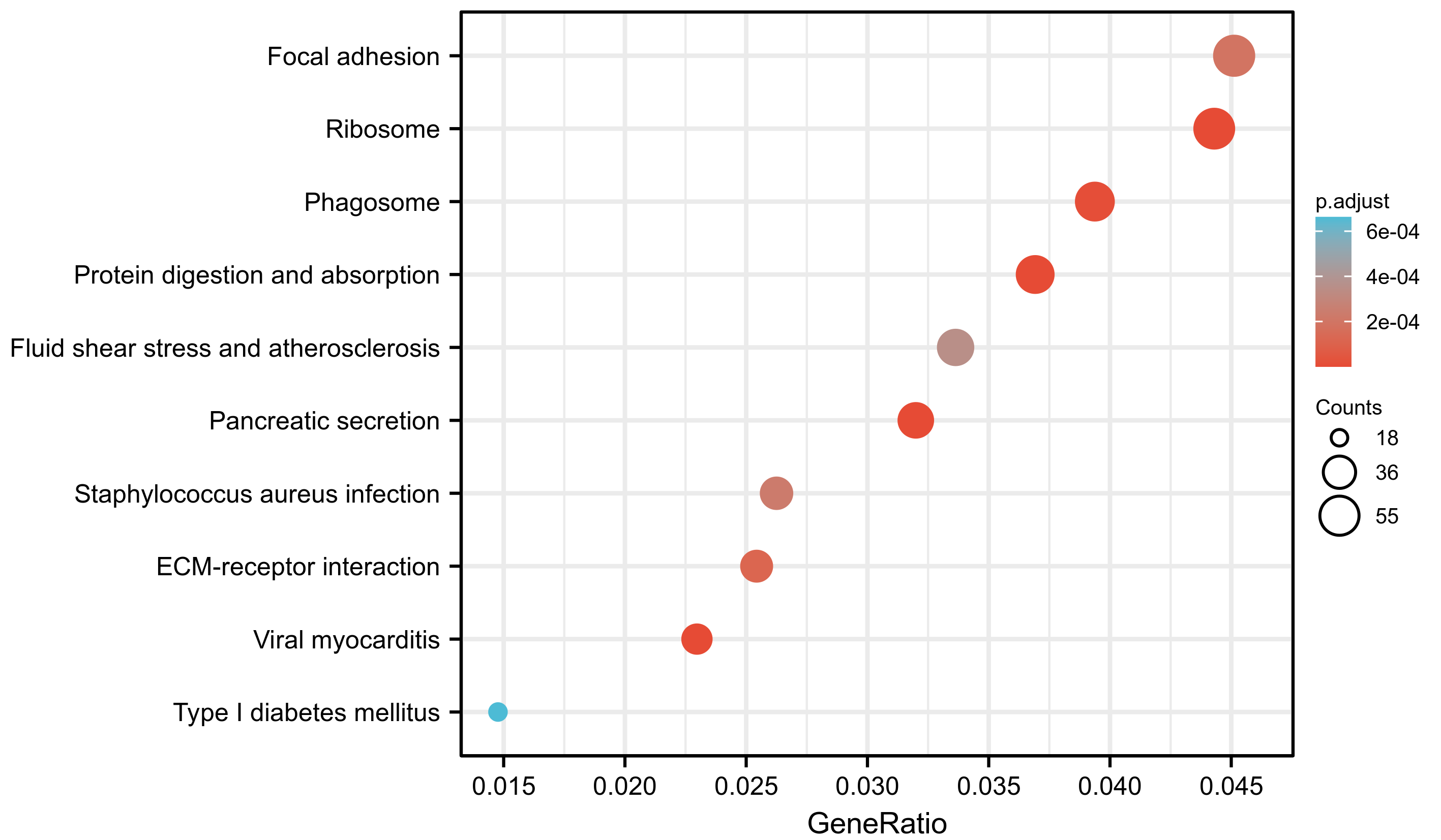


**Supplementary Figure 1** Bubble chart showing the enriched GO-BPs (A) and KEGG (B) pathways with all DEGs


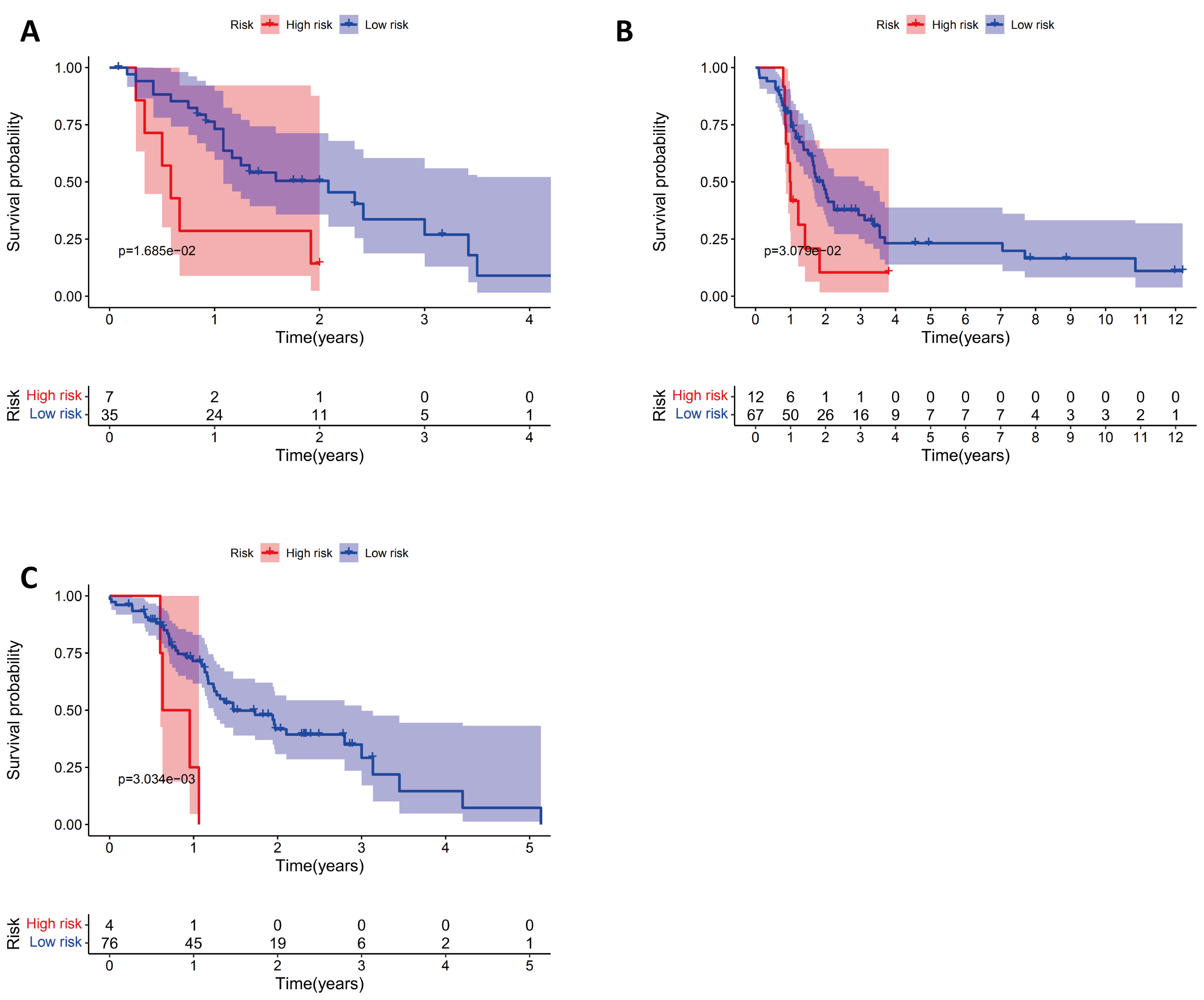


**Supplementary Figure 2** Survival Analysis of the PAAD Cohort. Kaplan-Meier analysis of the high-risk and low-risk groups of PAAD patients in the GDS4336 (A), GSE85916 (B), ICGC-PACA-AU (C).


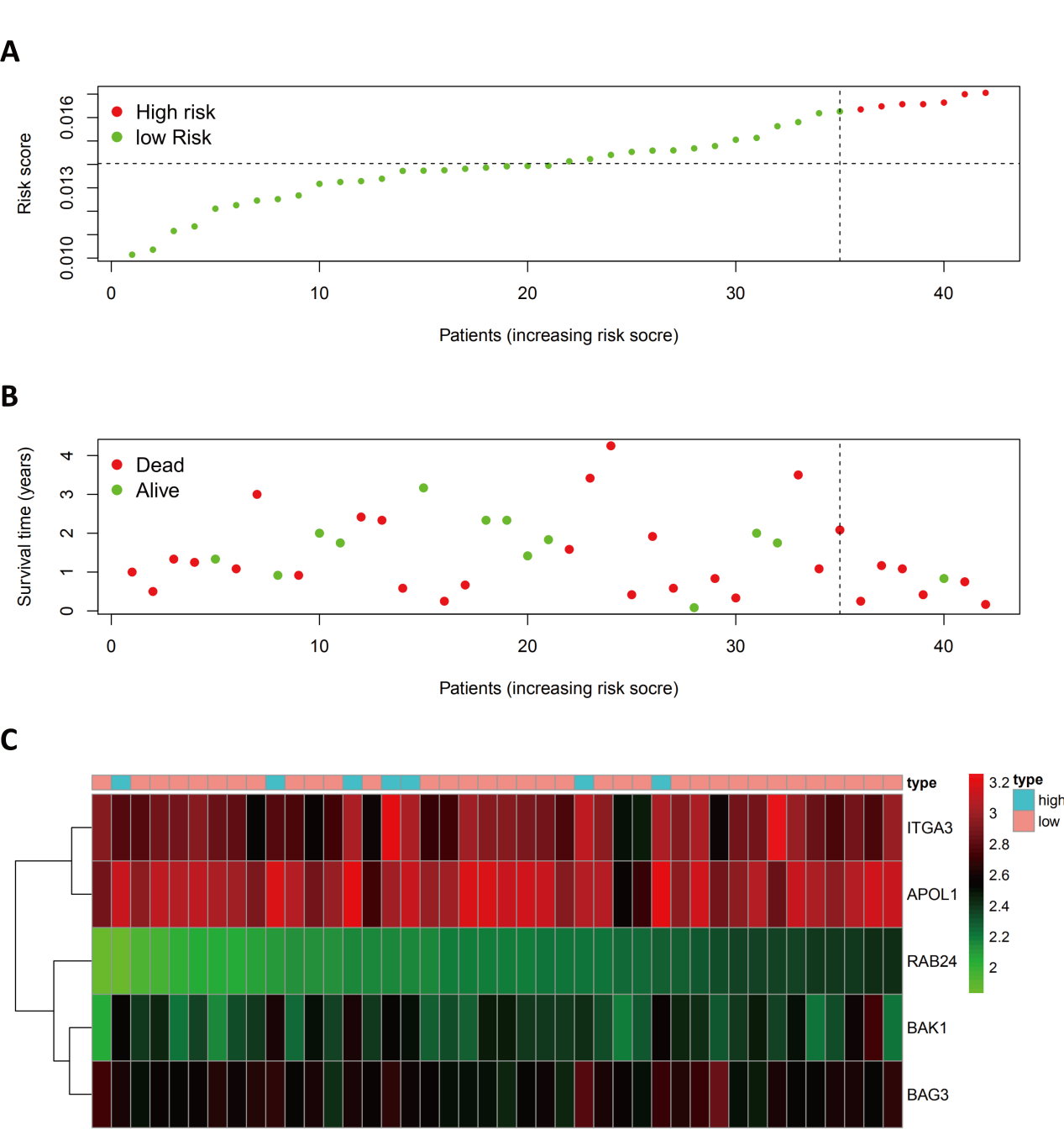


**Supplementary Figure 3** Evaluation of the Prognostic ARG Signature in the GDS4336 Dataset. (A) Distribution of the prognostic index. (B) Survival status of patients in the low- and high-risk groups. (C) Heat map of the expression profile of the included ARGs.


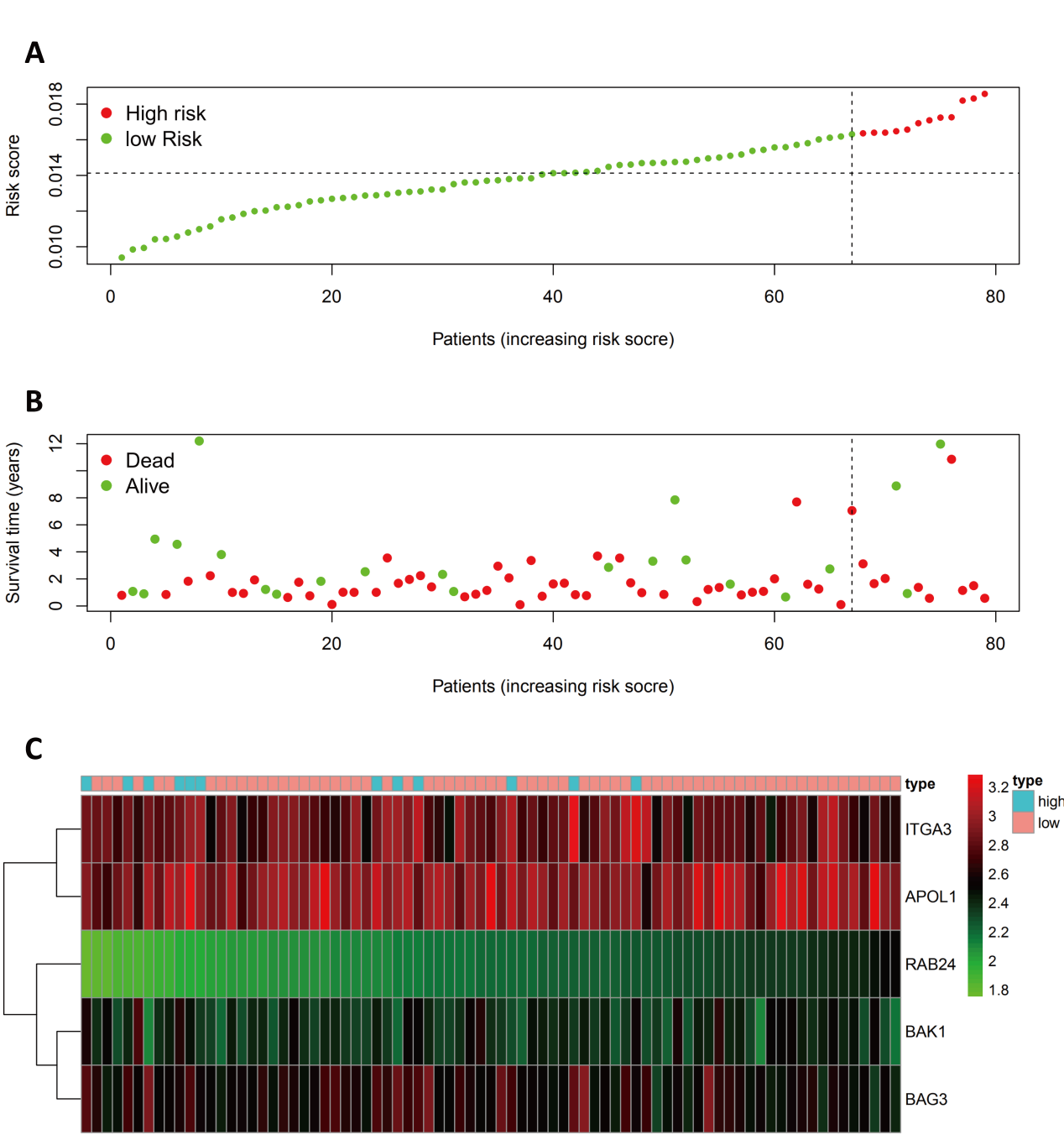


**Supplementary Figure 4** Evaluation of the Prognostic ARG Signature in the GSE85916 Dataset. (A) Distribution of the prognostic index. (B) Survival status of patients in the low- and high-risk groups. (C) Heat map of the expression profile of the included ARGs.


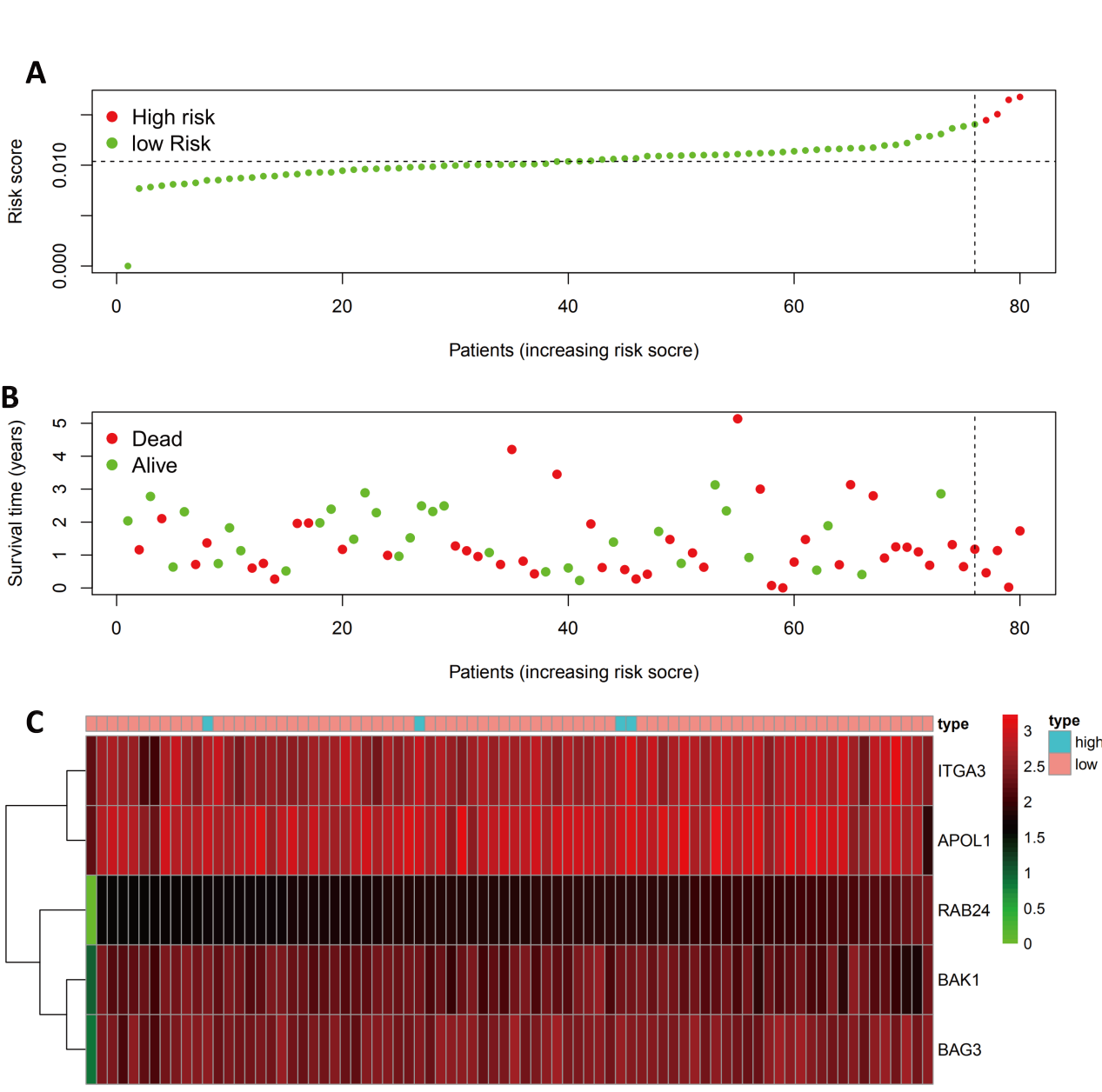


**Supplementary Figure 5** Evaluation of the Prognostic ARG Signature in the ICGC-PACA-AU Dataset. (A) Distribution of the prognostic index. (B) Survival status of patients in the low- and high-risk groups. (C) Heat map of the expression profile of the included ARGs.

**Supplementary Figure 6** The baseline data of immunohistochemical images

BAK1

Tumor


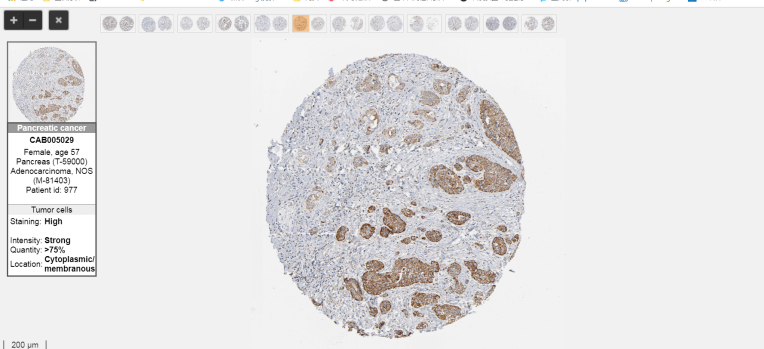


Normal


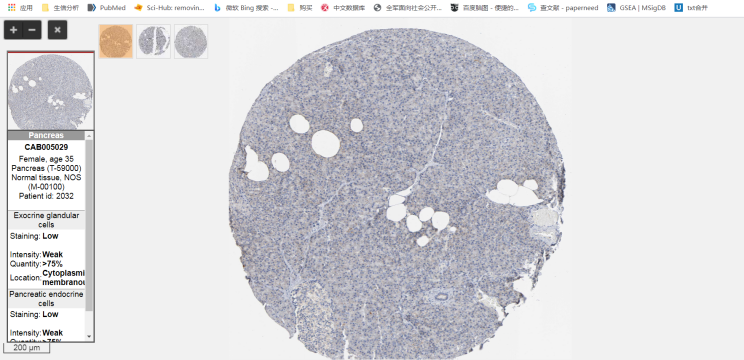


ITGA3

Tumor


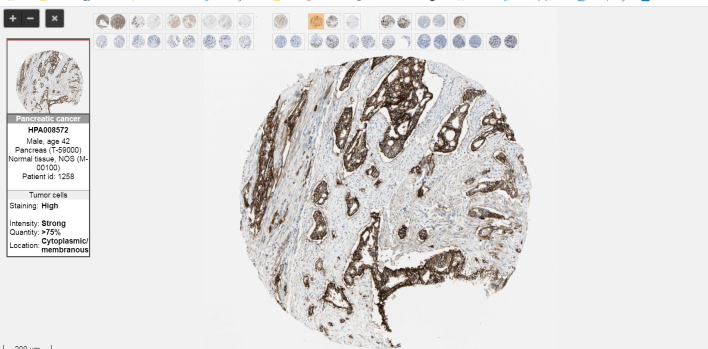


Normal


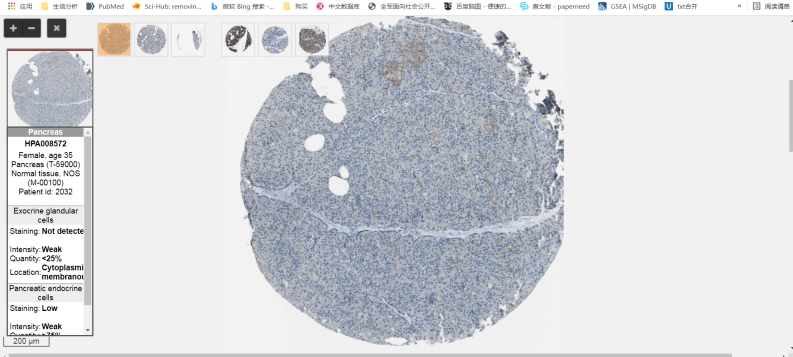


BAG3

Tumor


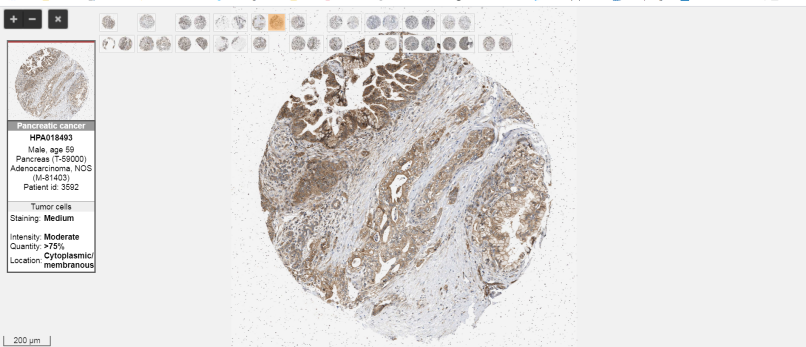


Normal


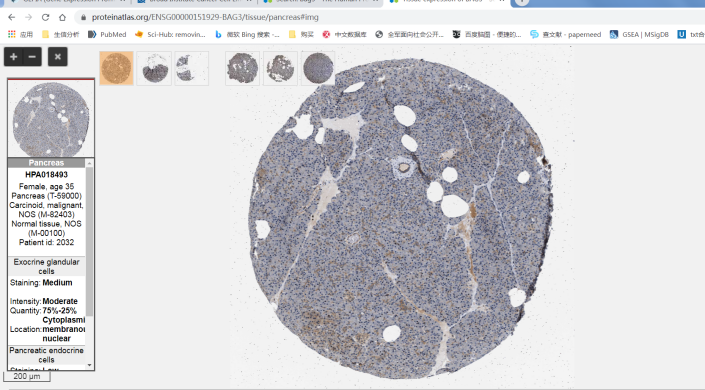


APOL1

Tumor


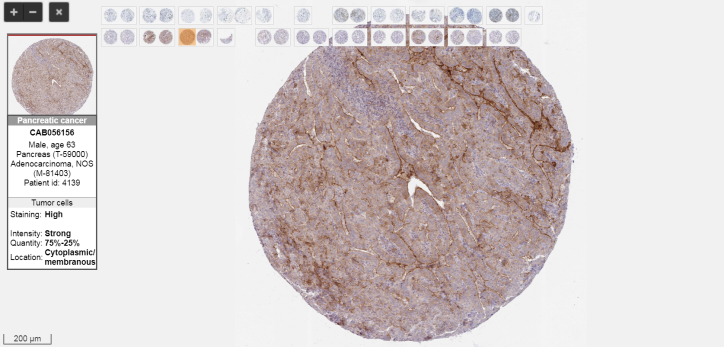


Normal


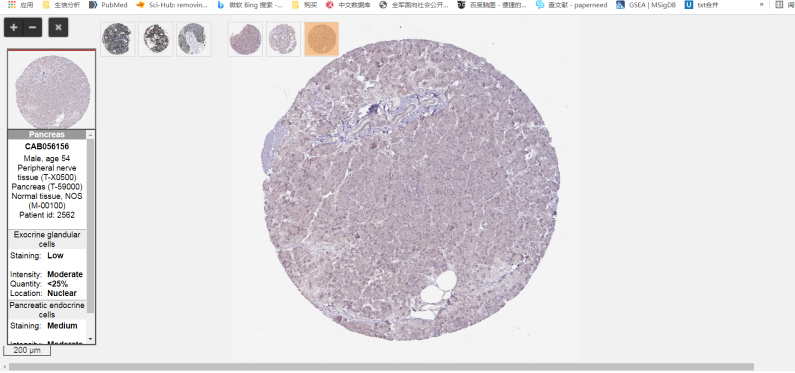

Supplement: Supplementary file 1 — Supplementary Information 1. [file 41598_2022_11050_MOESM1_ESM.docx]
